# Perturbation and resilience of the gut microbiome up to 3 months after β-lactams exposure in healthy volunteers suggest an important role of microbial β-lactamases

**DOI:** 10.1186/s40168-023-01746-0

**Published:** 2024-03-12

**Authors:** Camille d’Humières, Margot Delavy, Laurie Alla, Farid Ichou, Emilie Gauliard, Amine Ghozlane, Florence Levenez, Nathalie Galleron, Benoit Quinquis, Nicolas Pons, Jimmy Mullaert, Antoine Bridier-Nahmias, Bénédicte Condamine, Marie Touchon, Dominique Rainteau, Antonin Lamazière, Philippe Lesnik, Maharajah Ponnaiah, Marie Lhomme, Natacha Sertour, Savannah Devente, Jean-Denis Docquier, Marie-Elisabeth Bougnoux, Olivier Tenaillon, Mélanie Magnan, Etienne Ruppé, Nathalie Grall, Xavier Duval, Dusko Ehrlich, France Mentré, Erick Denamur, Eduardo P. C. Rocha, Emmanuelle Le Chatelier, Charles Burdet, for the PrediRes study group

**Affiliations:** 1 Université Paris Cité, IAME, INSERM, Paris, F-75018 France; 2 Institut Pasteur, Université Paris Cité, CNRS UMR3525, Microbial Evolutionary Genomics, Paris, 75015 France; 3 Institut Pasteur, Université Paris Cité, INRAE, USC2019, Unité Biologie Et Pathogénicité Fongiques, Paris, F-75015 France; 4 Université Paris-Saclay, INRAE, MetaGenoPolis, Jouy-en-Josas, F-78350 France; 5 INSERM UMR-S 1166, Institute of Cardiometabolism and Nutrition, Sorbonne Université, Hôpital Pitié-Salpêtrière, Paris, F-75013 France; 6 ICANomics, Foundation of Innovation in Cardiometabolism and Nutrition (IHU ICAN), Paris, F-75013 France; 7 Sorbonne Université, INSERM U938, Centre de Recherche Saint-Antoine, Paris, F-75012 France; 8 Institut Pasteur, Université Paris Cité, Bioinformatics and Biostatistics Hub, Paris, F-75015 France; 9 AP-HP, Département d’Epidemiologie, Biostatistique and Recherche Clinique, Hôpital Bichat, Paris, F-75018 France; 10 Dipartimento di Biotecnologie Mediche, Università di Siena, Siena, I-53100 Italy; 11 AP-HP, Unité de Parasitologie-Mycologie, Service de Microbiologie Clinique, Hôpital Necker-Enfants-Malades, Paris, F-75015 France; 12 AP-HP, Laboratoire de Bactériologie, Hôpital Bichat, Paris, F-75018 France; 13 AP-HP, Centre d’Investigation Clinique, INSERM CIC 1425, Hôpital Bichat, Paris, F-75018 France; 14 AP-HP, Laboratoire de Génétique Moléculaire, Hôpital Bichat, Paris, F-75018 France; 15 University College London, Institute for Neurology, London, UK

**Keywords:** Antibiotics, Human gut microbiota, Metagenomics, Metabolomics, Resilience, β-lactamase

## Abstract

**Background:**

Antibiotics notoriously perturb the gut microbiota. We treated healthy volunteers either with cefotaxime or ceftriaxone for 3 days, and collected in each subject 12 faecal samples up to day 90. Using untargeted and targeted phenotypic and genotypic approaches, we studied the changes in the bacterial, phage and fungal components of the microbiota as well as the metabolome and the β-lactamase activity of the stools. This allowed assessing their degrees of perturbation and resilience.

**Results:**

While only two subjects had detectable concentrations of antibiotics in their faeces, suggesting important antibiotic degradation in the gut, the intravenous treatment perturbed very significantly the bacterial and phage microbiota, as well as the composition of the metabolome. In contrast, treatment impact was relatively low on the fungal microbiota. At the end of the surveillance period, we found evidence of resilience across the gut system since most components returned to a state like the initial one, even if the structure of the bacterial microbiota changed and the dynamics of the different components over time were rarely correlated. The observed richness of the antibiotic resistance genes repertoire was significantly reduced up to day 30, while a significant increase in the relative abundance of β-lactamase encoding genes was observed up to day 10, consistent with a concomitant increase in the β-lactamase activity of the microbiota. The level of β-lactamase activity at baseline was positively associated with the resilience of the metabolome content of the stools.

**Conclusions:**

In healthy adults, antibiotics perturb many components of the microbiota, which return close to the baseline state within 30 days. These data suggest an important role of endogenous β-lactamase-producing anaerobes in protecting the functions of the microbiota by de-activating the antibiotics reaching the colon.

Video Abstract

**Supplementary Information:**

The online version contains supplementary material available at 10.1186/s40168-023-01746-0.

## Background

The Human gut microbiome is composed of a variety of archaea, bacteria, viruses, fungi and protozoa, which have a complex relationship with their host, from mutualism or commensalism to pathogenesis [1]. Although the bacterial microbiota is relatively stable over time in healthy subjects, several factors can modify its composition, including age, lifestyle [2, 3] or the use of medication such as antibiotics, which are often absorbed or excreted through the digestive tract [4]. Even after parenteral administration, a variable fraction of the administered dose is indeed eliminated via the biliary tract. The resulting impact on the gut microbiota varies according to the fractional intestinal elimination [5].

Antibiotics are known to disrupt the structure of the bacterial microbiota at different levels (from phyla to strains), and the resilience of the latter results in a partial return to the pre-treatment state [6, 7]. Antibiotics induce selection for resistance in commensal and pathogenic bacteria, contributing to the dissemination of resistant bacterial strains in the environment [8, 9]. Given the high density and diversity of bacteria in the gut, it has been suggested that the gut plays a key role in the development and spread of bacterial resistance to antibiotics [10]. In addition to bacteria, numerous bacterial viruses (bacteriophages or phages), fungi and protozoa are present in the gut [11–13]. They may be indirectly affected by antibiotics, as competition for resources and predation leads to a complex network of interactions.

To evaluate the consequences of gut exposure to antibiotics, one must understand their long-term effects on healthy individuals. This avoids the interference of the effects of pathologies with the outcome of the bacteria-antibiotic interactions. The study of this perturbation requires the investigation of the multiple components describing the complex gut system. This includes biotic variables such as the population of specific bacteria of interest (e.g. Enterobacterales that include many pathogens), bacterial predators (phages) [12] and competitors (fungi). Other key insights are obtained by the study of metabolites (small molecules < 1500 Da), which are intermediate or end products of cell metabolism [14]. These metabolites are produced by the host, by the microorganisms or external sources, and can be co-metabolised. From the broadest to the most specific, two classic examples of host-gut microbiome co-metabolism are (i) the biosynthesis of primary bile acids from cholesterol by the host and their subsequent deconjugation, dehydroxylation, dehydrogenation and epimerisation by the gut microbiome [15], and (ii) the conversion of cholesterol into several microbial metabolites, of which coprostanol is by far the most important [16] influencing host cholesterol level [17]. While most of these variables have been shown to be important in delineating the effect of antibiotics on the gut [18–20], there is a lack of understanding of how they are associated and interact.

We recently conducted the CEREMI clinical trial [21], a study including 22 healthy subjects to understand and compare the impact of two intravenous β-lactam antibiotics, ceftriaxone and cefotaxime, on the intestinal microbiota, following standard clinical doses administered for 3 days. Our previous analysis of 16S rRNA gene sequences showed that both antibiotics had a marked impact on the composition of the gut microbiota, but no significant differences were observed between the two antibiotics, suggesting they have the same effects. Of note, only two subjects had detectable faecal concentrations of antibiotics, suggesting antibiotic degradation by β-lactamases produced by anaerobes from the gut while they reach the colon [22]. Here, we analysed these stool samples to gain insights into the perturbation of the gut system at multiple levels. For this, we coupled shotgun sequencing methods, targeted and untargeted metabolomics approaches with phenotypic and genotypic targeted analyses of the faecal content. We focused on the bacterial, phage and fungal components of the community, along with the metabolite composition and β-lactamase activity of the stool content. We assessed the perturbations induced by both antibiotics and studied the correlations between them. The same analysis was performed for resilience, i.e. return to baseline state. We then evaluated whether the baseline microbiota status is associated with protection from perturbation and/or resilience following antibiotic administration. This revealed a comprehensive view of the impact of antibiotics on the gut microbiota.

## Results

### A multi-organism/multi-omic study of the effect of antibiotics on the gut microbiota of healthy volunteers

**Fig. 1:**
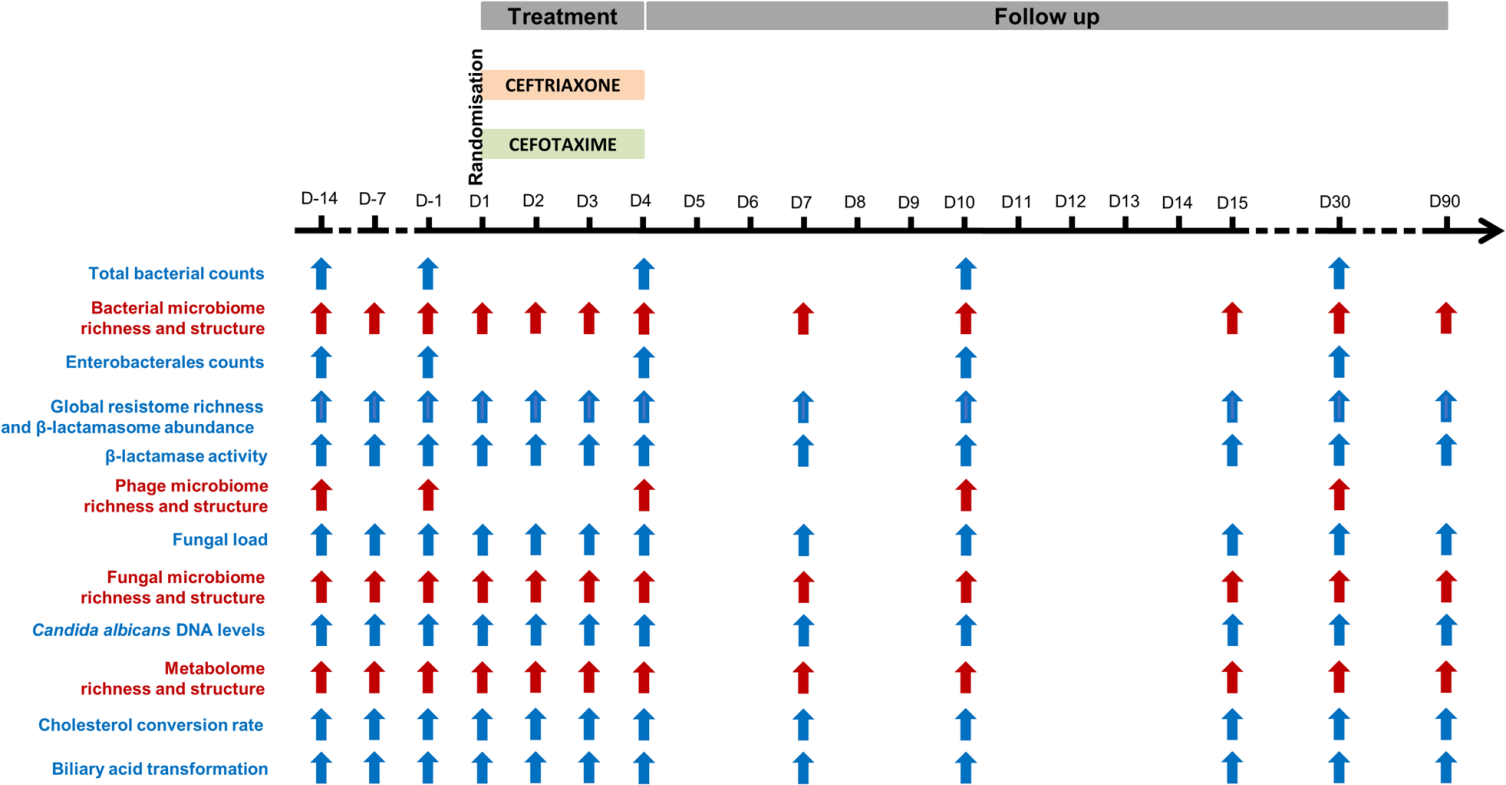
Design and sampling times of the CEREMI clinical trial. Systems depicted in red were classified as high dimensionality, and systems depicted in blue as low dimensionality

We administered to 22 healthy volunteers a standard 3-day course of intravenous β-lactam antibiotics partially eliminated through the intestinal route (Fig. 1). We sampled the stools of subjects (i) before (ii) during and (iii) after (up to day 90) the antibiotic treatment. We used different untargeted and targeted approaches to analyse phenotypic and genotypic characteristics of the bacterial, phage and fungal components of the microbiota, as well as the metabolic composition and β-lactamase content of the stools.

The variables obtained from these analyses were classified as high dimensional variables (metagenomic analyses of the bacterial, phage and fungal microbiomes, and metabolome) or low dimensional variables (observed richness of the bacterial, phage and fungal microbiomes, observed richness of the Antibiotic resistance genes (ARGs) repertoire and metabolome, relative abundance of the β-lactamasome, total bacterial counts, β-lactamase activity, fungal and *Candida albicans* DNA levels, cholesterol conversion into coprostanol and biliary acid transformation rates, see Fig. 1).

### Variability of the gut microbiota components before antibiotic treatment

We first studied the between and within subjects variability of low dimensional variables’ before antibiotics administration (Table 1), using a linear mixed-effect model treating subjects as random effects.

**Table 1:** Variability of the studied gut microbiota and stool components before antibiotic treatment administration in the 22 healthy volunteers included in the CEREMI trial

Component	*n*	Mean	Between subjects		Within subjects	
			Standard deviation	CV (%)	Standard deviation	CV (%)
Bacterial microbiota						
Bacterial counts (log_10_ CFU/g)	22	11.3	0.19	1.7	0.14	1.2
Bacterial microbiome richness (MGS/g)	22	269.7	64.69	24.0	16.01	5.9
Enterobacterales counts (log_10_ CFU/g)	22	7.5	1.37	18.3	1.00	13.3
Resistance						
Global resistome richness (copies/g)	22	820.1	159.50	19.5	69.75	8.5
β-lactamasome abundance (log_10_)	22	− 0.8	0.03	3.8	0.02	2.5
β-lactamase activity (log_10_ nmol/min.g)	22	1.2	0.59	49.2	0.20	16.7
Phage microbiota						
Phage microbiome richness (contigs/g)	21	1226.6	271.81	22.2	91.37	7.5
Fungal microbiota						
Fungal load (log_10_)	21	− 5.2	0.72	13.9	1.27	24.4
Fungal microbiome richness (fungal OTUs/g)	22	25.6	4.77	18.6	9.13	35.7
*C. albicans* DNA concentration (log_10_)	21	− 5.0	0.81	16.2	0.47	9.4
Metabolome						
Metabolome richness (chemical species/g)	22	1472.7	< 0.01	0.0	98.85	6.7
Cholesterol conversion rate (log_10_)	22	− 0.6	1.03	171.7	0.49	81.7
Bile acids transformation capacity (log_10_)	22	− 0.1	0.09	90.0	0.06	60.0

*n* Number of subjects with available data, *CV* Coefficient of variation

Most variables had a higher variability between subjects than within subjects. The bacterial microbiota appeared to be relatively stable within a subject, while the observed richness of the bacterial microbiome and counts of Enterobacterales had a variability of approximately 20% between subjects. In line with these observations, the phage microbiota, whose composition is expected to be related to that of the bacterial microbiota, had similar variability values.

Interestingly, the β-lactamasome abundance exhibited a very small between-subject variability (4%), while the β-lactamase activity had a relatively high variability (approximately 49%). The ARG repertoire was stable within individuals.

The fungal microbiota exhibited a high variability, especially within the subject. Overall, the specific metabolic functions of cholesterol conversion into coprostanol or biliary acid transformation had the highest variability, while the observed richness in chemical species was very stable both between and within subjects.

### Differential perturbation of the gut microbiota by antibiotics

**Fig. 2:**
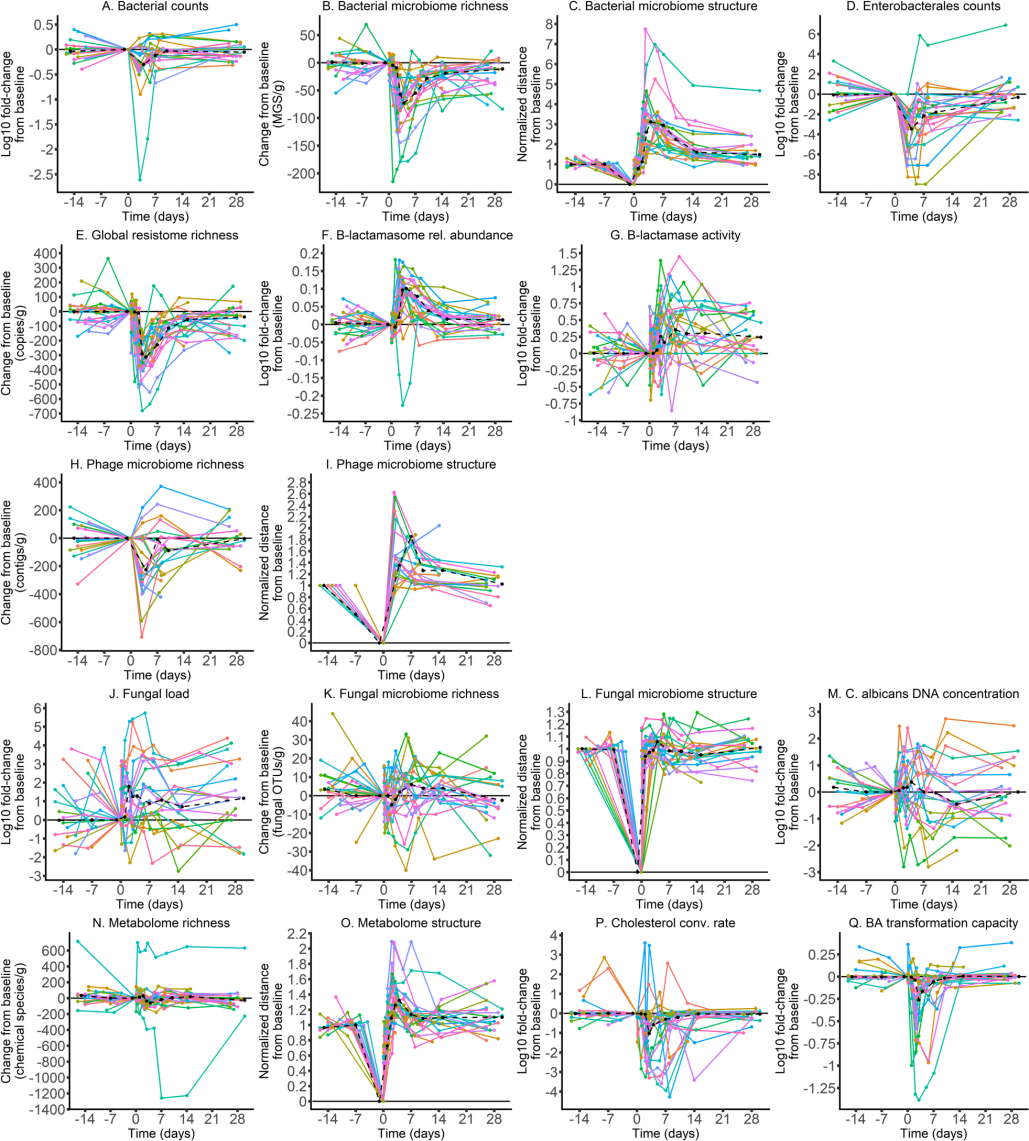
Evolution of the gut microbiota and stool components over time in the 22 healthy volunteers included in the CEREMI trial. For ‘low dimensionality’ systems, the log10 fold changes from baseline are presented (except for variables relative to the system’s richness which were not transformed), whereas for ‘high-dimensionality’ systems we depicted the normalised distance from baseline. In each graph, the *x*-axis indicates the time following antibiotic administration, whereas the *y*-axis corresponds to the change or distance from baseline (positive and/or negative) with the unit in brackets. Each individual is represented by a line with a specific color. Cholesterol conv. rate, Cholesterol conversion rate; BA, Bile acids; rel., relative

We then enquired about the perturbation that followed antibiotic administration. The gut microbiota of most individuals was significantly disrupted over the 30 days following antibiotic administration (Fig. 2 and Supplementary Table S1). We found very few significant differences between individuals treated with ceftriaxone or cefotaxime (Supplementary Table S2), suggesting similar effects of the two antibiotics. After correction for multiple testing, no significant difference was observed (Supplementary Table S2). This fits our previous analysis [21] and led us to analyse together the two groups of individuals. The results of these analyses show that the impact of antibiotics differed markedly from one component to another (Supplementary Table S1).

Antibiotics do not affect fungi directly (Fig. 1J–M), but some fungal species might profit from the depletion of bacterial populations to proliferate. Punctual perturbations in the fungal load were observed up to day 30, with a global increase in the fungal load after antibiotic treatment (Fig. 1J). The concentration of *C. albicans* DNA was not significantly impacted at the studied timepoints (Fig. 1M and Supplementary Table S1) and antibiotic treatment had little impact on the variables describing the fungal microbiome structure. Notably, we found no effect of antibiotics on the structure nor on the observed richness of the fungal microbiome (Fig. 1L). Hence, the treatment seems to have had little impact on this component of the gut, at least at the time points studied here.

In contrast, and as previously observed using 16S sequences [21], the bacterial microbiome was very perturbed (Fig. 1A–D). Although the bacterial counts showed only a slight decrease after antibiotic treatment, the observed richness of the bacterial microbiome was markedly decreased, and its structure was profoundly disrupted, with perturbation being still significant at day 90. Counts of Enterobacterales were significantly reduced up to day 10, with a maximal reduction observed on day 4, just after the end of the antibiotic treatment (Fig. 1D). The observed richness of the phage microbiome also decreased following antibiotic treatment, although the perturbation resolved earlier than for the bacterial microbiome (before day 10) (Fig. 1H, I). Hence, the bacterial fraction of the microbiome, and its viral predators, were very much affected by antibiotic treatment.

The lack of antibiotics in the faeces and the high perturbation observed in the bacterial fraction suggest antibiotics degradation in the gut. To analyse this, we searched for antibiotic-resistance genes in the bacterial genomes using mustard [23]. We found that among the 19,061 antibiotic resistance determinants identified, 1823 (9.6%) were genes encoding for β-lactamases. According to Ambler classification, they were distributed as follows: 627 from class A, 463 from classes B1-B2, 463 from class B3, 181 from class C and 89 from class D. The observed richness of the ARGs repertoire was significantly reduced up to day 30 (Fig. 1E), while a significant increase in the relative abundance of β-lactamase encoding genes was observed up to day 10 (Fig. 1F), which was consistent with an increase in the β-lactamase activity of the microbiota and the lack of antibiotics in faeces (Fig. 1G).

Overall, these results indicate very different levels of perturbation caused by antibiotics on the components of the gut microbiota. This resulted in a minimal perturbation of the metabolome observed richness, whose composition was however profoundly disrupted and was still far from the initial state at day 90. Functions of the microbiota, such as the conversion rate of cholesterol into coprostanol and the capacity of the microbiota to transform primary bile acids into secondary bile acids, were temporarily reduced but seemed to return to baseline within 10 days (Supplementary Table S1). This suggests that despite a long-term perturbation of the structure of the microbiota and its biochemical composition, the functions of the microbiota are not lost and can be restored quickly following exposure to antibiotics. The same results were observed after correction for multiple testing (Supplementary Table S1).

### Correlation between maximal perturbations of the different gut microbiota components

**Fig. 3:**
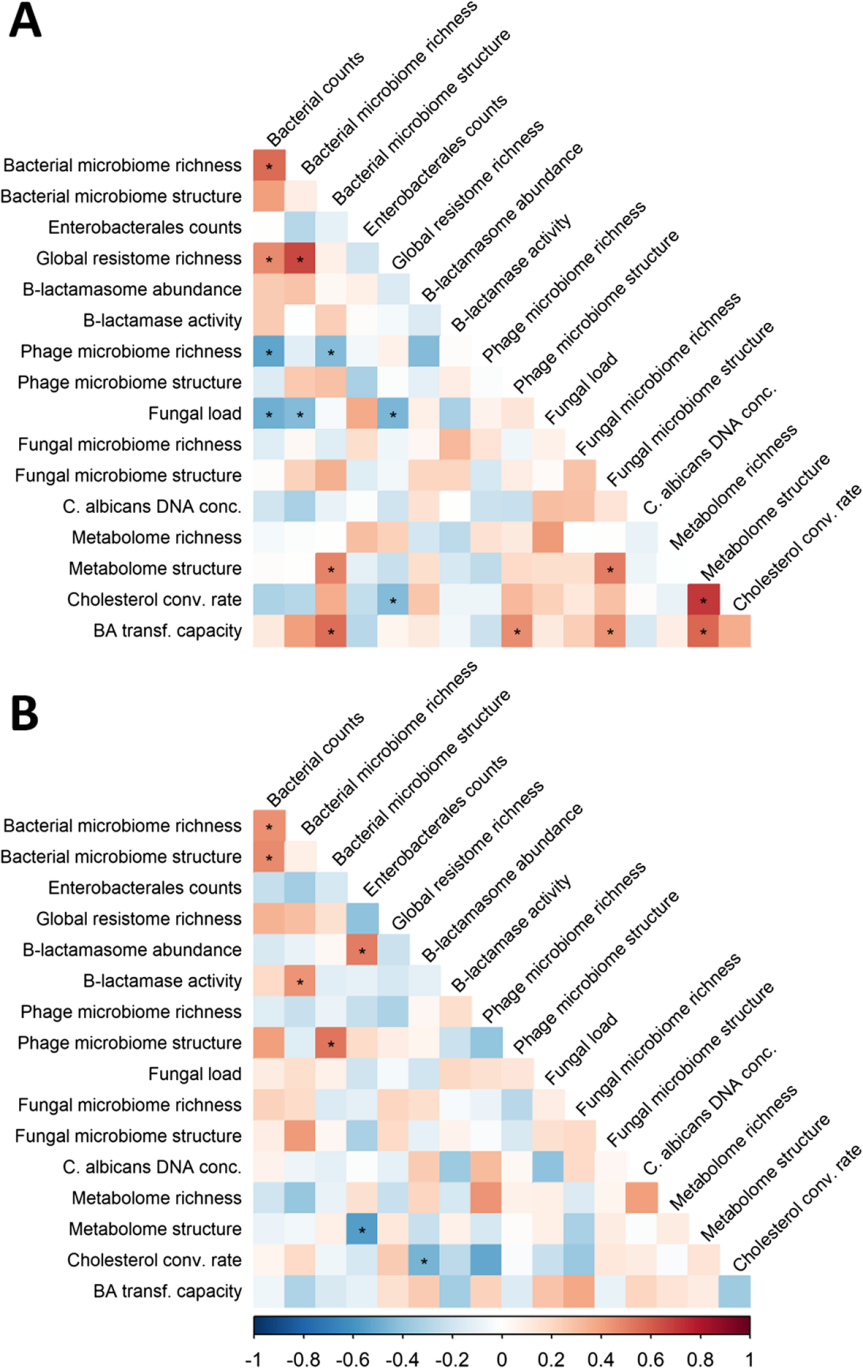
Correlograms of the maximal perturbations (**A**) and maximal resilience (**B**) of studied gut microbiota and stool components in the 22 healthy volunteers included in the CEREMI trial. The color intensity of the squares indicates the level of the Spearman correlation coefficient. Stars indicate a statistically significant Spearman’s correlation coefficient. Conc., concentration; Cholesterol conv. rate, Cholesterol conversion rate; BA transf. Capacity, Bile acids transformation capacity

To understand the relationship between each component’s perturbation following antibiotic exposure, we computed for each sample and at each sampling time a distance from the baseline to trace the evolution of the components over time. This distance was normalised by pre-treatment values to allow for comparisons between individuals and between variables. We defined the maximal perturbation as the maximal distance from baseline observed up to day 10. We then studied the correlations of these distances across variables to identify groups of variables that show similar patterns of perturbation. Among the 136 pairwise correlations, 16 (11.8%) were found to be statistically significant, even if their magnitude was moderate (maximal absolute value of 0.71) (Fig. 3A, and Supplementary Table S3). A cluster of significant positive correlations was observed between the maximal perturbations of the bacterial counts, bacterial microbiome observed richness and global ARGs repertoire observed richness. It was also positively correlated with the β-lactamasome abundance and the β-lactamase activity, although in these cases the values were not significant. This confirms the association between the level of perturbation in the bacterial component of the microbiota and that of antibiotic-resistance genes in the bacteria.

One might have expected an association between the maximal perturbation in the phage fraction of the microbiome and other variables, especially those associated with the bacterial composition. Intriguingly, more pronounced changes in the bacterial counts were significantly associated with a less pronounced perturbation in phage observed richness, i.e. when the bacterial counts decreased to a greater extent, we observed a lower change in the number of different phage contigs. One possibility is that phage induction by antibiotics, which has been described even at sub-inhibitory concentrations [24, 25], has a role in bacterial mortality that stabilises the absolute number of phages. If so, the expected decrease in phage associated with the decrease in bacterial populations by the action of antibiotics would be compensated by increased induction rates of the prophages, which would amplify bacterial death rates.

As expected, the maximal perturbation of the metabolome was correlated to the maximal perturbation of the cholesterol conversion rate into coprostanol, the bile acid transformation capacity, and the bacterial microbiome structure. Interestingly, the perturbation of the bile acid transformation capacity was correlated with the level of perturbation of the structure of all studied components, i.e. bacterial, phage and fungal.

Finally, we found no significant perturbation in the observed richness or the structure of the fungal microbiome. Accordingly, it was not correlated with the other perturbations. However, the maximal perturbation of the fungal load was negatively correlated with that of the total bacterial counts, suggesting a direct inverse association between perturbations at the bacterial and fungal scales. Hence, fungi thrive when bacterial populations are rarefied by antibiotics, which seems to leave the structure and observed richness of their population relatively unchanged. The abundance of fungi increases and is associated with significant changes in the metabolic functions of the microbiota.

In a sensitivity analysis accounting for the false discovery rate, the only correlation that remained significant was that between the perturbation of the metabolome structure and the cholesterol conversion rate (Supplementary Table S3).

### Correlation between resilience of the different gut microbiota components

Once perturbations subside, systems may return to a situation close to the initial one, which we refer to as resilience. For the analysis of resilience, we computed for each variable the minimal normalised distance from baseline observed on the samples collected between day 15 and day 90. We then made correlations across all variables as described above for perturbations. Overall, 7 (5.1%) of the 136 pairwise correlations were statistically significant, and all with a moderate magnitude (maximal absolute value of 0.55) (Fig. 3B and Supplementary Table S4). We found a cluster of positive correlations between the resilience of the bacterial microbiome observed richness and structure, of the β-lactamasome, of the β-lactamase activity, and of the bacterial counts. Even if only some pairwise correlations between these systems were significant, this suggests the existence of a group of variables with similar patterns of resilience associated with bacterial composition and protection from the agents producing the perturbation (antibiotics). Although their functional profile has not been established, the reported diversity of intestinal β-lactamases from bacterial species belonging to various phyla supports this assumption [23]. The resilience of the structure of the phage microbiome was also correlated with that of the bacterial microbiome, suggesting a tight association between the mechanisms of recovery of both variables. This may be caused by the arrest of prophage induction shortly after the end of antibiotic treatment, which would tightly link the recoveries of the populations of phages and bacteria.

In a sensitivity analysis accounting for the false discovery rate, no significant correlation was observed between the resiliences of the various parts of the microbiome (Supplementary Table S4).

### Relationship between baseline composition, maximal perturbation and resilience of the gut microbiota components

One would expect an increase in the frequency of antibiotic-resistance genes in bacteria following exposure to antibiotics because those encoding them are more likely to survive. Indeed, the content in antibiotic-resistance conferring genes was disrupted, with an increase of genes encoding for β-lactamases, even if this was also associated with a decrease of other ARGs (Fig. 2). These changes were followed by a significant increase in the β-lactamase activity of the microbiota.

**Fig. 4:**
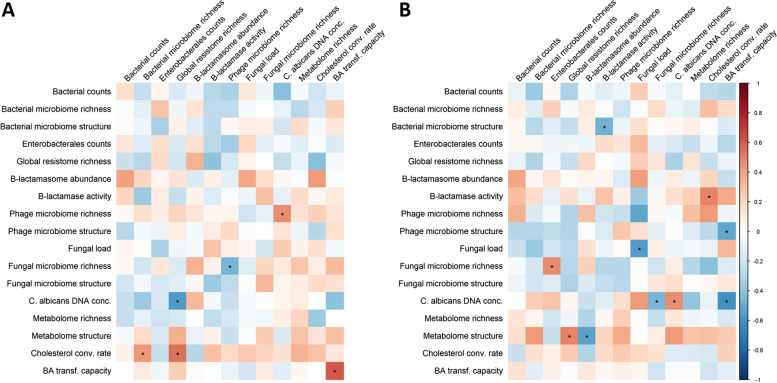
Relationship between the baseline characteristics and the maximal perturbations (**A**) and maximal resilience (**B**) of the studied gut microbiota and gut components in the 22 healthy volunteers included in the CEREMI trial. Baseline characteristics of the intestinal microbiota are presented in the top horizontal axis, while the maximal perturbations or resilience are presented in the left vertical axis. For the correlation between the baseline characteristics of the intestinal microbiota and the maximal perturbations, a positive correlation is interpreted as an increase in the level of perturbation when the baseline characteristic increases. For the correlation between the baseline characteristics of the intestinal microbiota and the maximal resilience, a negative correlation is interpreted as an increase in the level of resilience when the baseline characteristic increases. The colour intensity of the squares indicates the level of the Spearman correlation coefficient. Stars indicate a statistically significant Spearman’s correlation coefficient. Conc., concentration; Cholesterol conv. rate, Cholesterol conversion rate; BA transf. capacity, Bile acids transformation capacity

We then set out to evaluate how the initial state of the microbiota, including its content in β-lactamases encoding genes, might influence the antibiotic-induced perturbations. For this, we investigated the correlations between the initial composition of the gut microbiota (using the untransformed values of each variable at baseline) and the maximal perturbation of the variable among the 22 sampled volunteers, measured by the normalised distances from baseline. We observed few significant correlations and they had moderate absolute magnitudes (Fig. 4A). In particular, the relative abundance of β-lactamase encoding genes or the β-lactamase activity was not significantly associated with a reduction in the level of perturbation of any of the studied systems. This suggests that the baseline level in β-lactamases of the microbiota does not limit the perturbation induced by antibiotics. For reasons that are unclear at this stage, the perturbation of the cholesterol conversion rate into coprostanol was higher when the observed richness of the global ARGs repertoire at baseline was high. This might be an indirect effect due to the relation between the ARG repertoire and the bacterial microbiome. More expectedly, when the observed richness of resistance genes at the baseline was high, the increase in the frequency of *C. albicans* was lower. This suggests that bacteria more resistant to antibiotics could provide fewer niches for the expansion of the populations of *C. albicans*.

However, in a sensitivity analysis accounting for the false discovery rate, no significant correlation was observed between the baseline composition of the microbiome and its maximal perturbation (Supplementary Table S5).

The same type of analysis was performed to study the correlation between the initial composition of the gut microbiota and the maximal resilience of the gut components (Fig. 4B). We observed that the level of β-lactamase activity at baseline, but not that of the relative abundance of β-lactamase encoding genes, was positively correlated with the resilience of the bacterial microbiome and metabolome structures. The counts of Enterobacterales at baseline were negatively correlated with the resilience of the fungal microbiome observed richness. We also observed a cluster of correlations between variables associated with fungi: the baseline values of the fungal load, of the fungal microbiome observed richness and of the *C. albicans* DNA concentration and the resilience of the fungal load and *C. albicans* DNA concentration. This suggests that a complex and rich fungal microbiota might facilitate its restauration after a perturbation.

Of note, in a sensitivity analysis accounting for the false discovery rate, no significant correlation was observed between the baseline composition of the microbiome and its resilience (Supplementary Table S6).

### A focus on the rare subjects with antibiotics in the faeces

Ceftriaxone and cefotaxime are excreted in part through the intestinal route, which explains the perturbations found in this study, but only 2 of the 22 included subjects had detectable levels of antibiotics in faeces [21]. This is probably related to the ability of β-lactamases produced by bacteria from the gut to hydrolyse β-lactam antibiotics in the faecal content [26, 27].

The two subjects who had detectable beta-lactam residues in the faeces, subject #16 (from the ceftriaxone treatment group) had detectable concentrations between days 2 and 7 ranging between 5.0 µg/g and 93.7 µg/g while subject #3 (from the cefotaxime treatment group) had detectable concentrations of cefotaxime in faeces at day 4 (1.6 µg/g). Of note, both showed no detectable β-lactamase activity at baseline (Supplementary Table S7). These subjects also had among the lowest bacterial observed richness before antibiotic treatment, with a particularly low observed richness in resistance-conferring genes. Intriguingly, their β-lactamasome was among the most abundant among the study participants, suggesting that these abundant antibiotic-resistance genes were either not expressed or non-functional.

These two subjects exhibited different levels of perturbation following antibiotic treatment: despite the absence of β-lactamase activity at baseline, subject #3 was among those with the least altered gut microbiota, while subject #16 was among those with the most disrupted (Supplementary Figure S1). The analysis of these two subjects suggests that outcomes of antibiotic therapies can be quite variable and sometimes unexpected.

## Discussion

Here, we evaluated the impact of a short course of commonly used antibiotics in the hospital on the gut microbiota from healthy volunteers who had not been exposed to antibiotics for an extended period. We assessed this impact from several perspectives, analysing over 90 days the population dynamics of microorganisms, the antibiotic-resistance determinants, and some key metabolic functions, either using high throughput untargeted approaches or targeted tools. In the present report, we focused on global analysis, and an in-depth analysis of individual components will be presented elsewhere.

We observed that the dynamics of disturbance over time, followed by those of resilience, are correlated for only a small part of the studied components. This indicates that, although gut microbiota can be considered as a network of interactions, some features are behaving in a similar way whereas others are not.

As expected, we observed that bacterial and phage population structures were quickly disrupted, as were the bacterial counts. Return to baseline composition after antibiotic exposure has been found to range from a few weeks to months, according to the type of exposure and the methodology used to qualify the microbiota composition [7, 21, 28]. The effect of antibiotics on the bacterial populations of the gut microbiota has long been known [29, 30], yet the interest in the Human gut phage population is more recent, and the existence of a characteristic healthy gut phage population has recently been suggested [31]. A few studies investigated the disruption of the phage population following antibiotic administration [32, 33]. Here, we confirmed this disruption and the progressive return to a baseline state. Yet, contrary to a recent study where patients were treated against *Helicobacter pylori* and recovery of the phage community was slower than that of the bacteria [34], here this return occurred before that of the bacterial microbiome. The reasons for this difference are unclear, but could be due to differences in length and type of therapy, our sampling of many more time points, or the differences between healthy and other individuals. The faster stabilisation of the population of phages suggests than re-colonisation of the gut by bacteria may be affected by existing phage composition.

The changes in the frequency of antibiotic-resistance genes are particularly interesting to understand the impact of antibiotics in the promotion of resistant bacteria and/or the protection of the microbiota. We observed variations in the repertoire of antibiotic-resistance genes, and in particular at day 4 a decrease of the observed global ARGs repertoire richness simultaneous with an increase of the relative abundance of β-lactamase encoding genes and the β-lactamase activity. This is coherent with previous findings following the administration of a cocktail of 3 broad-spectrum antibiotics (meropenem, gentamicin, and vancomycin) [7]. Likely, these variations are due to the loss of bacteria that encode ARGs other than β-lactamase and the persistence of bacteria that do encode β-lactamase. Intriguingly, the changes in the abundance of β-lactam resistance genes or β-lactamase activity were not associated with the changes observed in the bacterial component of the microbiota. It was however associated with an increased resilience of the metabolomic content of the microbiota, suggesting that the functional resilience of the microbiota following antibiotic-induced perturbation might be enhanced by β-lactamases produced by bacteria in the gut. A recent study of healthy volunteers receiving widely prescribed antibiotics (ciprofloxacin, clindamycin, minocycline and amoxicillin) showed minimal microbiome perturbance under the β-lactam amoxicillin [35], in agreement with our hypothesis.

Although the abundance of fungi increased after antibiotic exposure, the structure of the fungal microbiome was not strongly affected and we could not detect any changes in *C. albicans* DNA levels after antibiotics administration at the studied timepoints. A more detailed analysis of the mycobiota conducted by our group on the 22 subjects suggested that *C. albicans* levels increased immediately after antibiotic administration in some subjects, this increase being subject-dependant and relying largely on the variations in β-lactamase activity observed after the antibiotic treatment [36]. The perturbation of the fungal load and *C. albicans* DNA levels after antibiotics was previously reported in mice, where the fungal burden was increased after bacterial elimination by antibiotics [37]. Furthermore, antibiotics are required to allow *C. albicans* gut colonisation in mice [38]. These results suggest that *C. albicans* growth could be efficiently prevented by specific bacteria residing in the human gut [39]. The decrease of specific bacterial populations induced by antibiotics would then open the way for the overgrowth of *C. albicans*. In agreement with this hypothesis, we observed that the perturbation of *C. albicans* DNA level was negatively correlated with the bacterial microbiome observed richness at baseline. Hence, the lack of diversity in the bacterial fraction and antibiotics favors the expansion of *C. albicans.*

Alterations of the faecal metabolome structure followed the trends observed for bile acid and sterol profiles. Disruption of both faecal bile acid and cholesterol metabolisms by antibiotics was previously reported [40, 41]. In particular, this led to an enrichment of faecal cholesterol and primary conjugated bile acids and the loss of coprostanol and secondary bile acids [42]. Of interest, this study found a particularly weak correlation between the largest perturbations observed in ‘BA conversion capacity’ and ‘cholesterol conversion rate’. This intriguing observation raises the possibility that these fundamental processes controlled by bile salt hydrolase and cholesterol reductase are actually controlled by bacterial strains acting independently. This longitudinal dataset also confirmed the established association between bile salt hydrolase and bacterial microbiome structure, illustrated by a clear correlation between perturbations in ‘bile acid conversion capacity’ and changes in the structure of bacterial populations. This dataset also reveals complex interrelationships between these complex ecological dynamics of bacterial, fungal, and phage structures and their bile acid hydrolysis capacity, which represents a new area of investigation.

These observations should be tempered by the fact that, after correction for multiple tests, most of the associations observed were not significant. This could be linked to a lack of power, as the CEREMI trial was not designed for such precise analysis at the metagenomic level.

However, our data suggest collectively that metabolomic signatures following antibiotic treatment are primarily related to the dynamics of disruption of gut-resident structures (microbiome, phage, and fungi) over time rather than the dynamics of their recovery, consistent with their cellular or viral origins. Moreover, the baseline characteristics of metabolomic features may determine the maximal disruption of the ARGs repertoire observed richness and β-lactamasome abundance revealing potential inter-domain connection.

## Conclusions

Antibiotics affect multiple aspects of the gut microbiota and stool composition of healthy individuals. This leads to a change in the metabolites present in the gut, and noticeably of cholesterol and bile acids. However, the perturbation of the system does not irreversibly change it. Instead, we observed resilience at 30 days, and a positive relationship between the baseline levels of β-lactamase activity in the gut and the structure of the metabolome.

These data indicate that a normal microbiota is able to absorb the antibiotic stress, probably thanks to the β-lactamasome of anaerobes [23]. This underlines their importance in protecting the functions of the microbiota against the deleterious effects of antibiotics and paves the way for the use of either cephalosporinases released in the colon by *Bacteroides* sp [27] or powerful antibiotic adsorbent acting in the late ileum as activated charcoal [28] to combat antibiotic-induced microbial dysbiosis.

## Methods and materials

### Study population and sample collection

We analysed the samples collected during the CEREMI trial (ClinicalTrials.gov identifier NCT02659033), a prospective, randomised open-label clinical trial conducted at the Clinical Investigation Center of the Bichat-Claude Bernard Hospital (Paris, France) from March 2016 to August 2017. The trial was sponsored by Assistance Publique-Hȏpitaux de Paris and approved by French Health Authorities and by the Independent Ethics Committee Ȋle-de-France-1. All procedures were conducted in compliance with good clinical practice and the Declaration of Helsinki. Full details of the trial have been reported elsewhere [21].

Briefly, healthy volunteers of both genders aged between 18 and 65 years old without exposure to antibiotics in the preceding 3 months nor a history of hospitalisation in the last 6 months were prospectively included after obtention of their informed consent. A total of 22 healthy volunteers were randomly assigned (1:1 ratio) to receive for 3 days either 1 g of ceftriaxone once a day (*n* = 11) or 1 g of cefotaxime three times a day (*n* = 11). Antibiotic treatment was administered as 30-min intravenous infusions. For each volunteer, 12 faecal samples were collected (Fig. 1): before the beginning of treatment at days − 15, − 7 and − 1; every day during treatment at days 1, 2 and 3, and after the end of treatment at days 4, 7, 10, 15, 30 and 90.

### Bacterial counts

Sample collected at days 15, − 1, 4, 10 and 30 were analysed to determine the total bacterial counts (Fig. 1). Aliquots containing 200 mg of faeces were diluted 200,000 times in a physiological solution (8.5 g/L NaCl). Samples were filtered for debris removal from faecal solutions using a sterile syringe filter (pore size 5 μm; Sartorius Stedim Biotech GmbH, Göttingen, Germany). Then, 1 mL of the microbial cell suspension obtained was stained with 1 μL SYBR Green I (1:100 dilution in dimethylsulfoxide; shaded 15 min incubation at 37 °C; 10,000 concentrate, Thermo Fisher Scientific, Waltham, MA, USA). The flow cytometry analysis of the microbial cells present in the suspension was performed using a C6 Fortessa flow cytometer (BD Biosciences, Franklin Lakes, NJ, USA). Fluorescence events were monitored using the FITC filter 505LP 530/30 nm and perCP filter 635LP 695/40 nm optical detectors. Forward and sideways-scattered light was also collected. The BD Accuri CFlow software was used to the gate and separate the microbial fluorescence events on the FL1–FL3 density plot from the faecal sample background. A threshold value of 200 was applied to FSC/SSC light. The gated fluorescence events were evaluated on the forward–sideways density plot, to exclude remaining background events and to obtain an accurate microbial cell count.

### Metagenomic analysis of the bacterial microbiome

All samples were analysed through shotgun sequencing for bacterial microbiome analysis.

#### DNA extraction of stool samples and shotgun sequencing

DNA extraction from aliquots of all faecal samples was performed following IHMS SOP P7 V2 (Fig. 1) [43]. DNA was quantitated using Qubit Fluorometric Quantitation (ThermoFisher Scientific, Waltham, MA, USA) and qualified using DNA size profiling on a Fragment Analyzer (Agilent Technologies, Santa Clara, CA, USA). Three µg of high molecular weight DNA (> 10 kbp) was used to build the library. Shearing of DNA into fragments of approximately 150 bp was performed using an ultrasonicator (Covaris, Woburn, MA, USA) and DNA fragment library construction was performed using the Ion Plus Fragment Library and Ion Xpress Barcode Adapters Kits (ThermoFisher Scientific, Waltham, MA, USA). Purified and amplified DNA fragment libraries were sequenced using the Ion Proton Sequencer (ThermoFisher Scientific, Waltham, MA, USA), generating 22.2 ± 1.8 million reads of 150 bp (on average) per sample.

#### Microbial gene count table

To create the gene count table, the METEOR software was used [44]: first, reads were filtered for low quality by AlienTrimmer [45]. Reads that aligned with the human genome (identity > 95%) were also discarded. The remaining reads were trimmed to 80 bases and aligned to the Integrated Gut Catalogue 2 (IGC2) [46] with a mapping rate of 81.5 ± 6%, comprising 10.4 million genes, using Bowtie2 [47]. The unique mapped reads (reads mapped to a unique gene in the catalogue) were attributed to their corresponding genes. The shared reads (reads that mapped with the same alignment score to multiple genes in the catalogue) were attributed according to the ratio of their unique mapping counts of the captured genes. The resulting count table was further processed using the R package MetaOMineR v1.31 [48]. To decrease technical bias due to different sequencing depths and avoid any artifacts of sample size on low-abundance genes, read counts were ‘rarefied’ using 20 M high-quality reads (a threshold chosen to include all samples) using a random sampling procedure without replacement. The downsized matrix was finally normalised dividing gene read counts per gene length × 100, as a proxy of gene coverage. Since gut microbiota has been found to be enriched in species from the oral cavity upon antibiotic treatment [49], and in order to improve the metagenomes annotation, the same process was repeated on an oral microbiota catalogue of 8.4 million genes [50]. Counts matrix were then merged to get a single matrix.

#### Metagenomic Species (MGS) profiles

The IGC2 and the oral catalogues were organised into 1990 and 853 Metagenomic Species (MGS, cluster of co-abundant genes), respectively, using MSPminer [50–52]. After removing duplicated MGS (i.e. MGS present in both catalogues), we were left with 2741 MGS. The relative abundance of an MGS was computed as the mean abundance of its 100 ‘marker’ genes (that is, the genes that correlate the most altogether). If less than 10% of ‘marker’ genes were seen in a sample, the abundance of the MGS was set to 0. MGS abundance profiles were finally normalised to estimate the proportion of each species in the microbiota (sum of all species abundance = 1).

Bacterial microbiome observed richness of each sample was evaluated as the number of unique species (MGS) identified. Bacterial microbiome structure is evaluated according to species abundance.

### Determination of the Enterobacterales counts

During the CEREMI trial, faecal samples from all volunteers (Fig. 1) were stored at 4 °C after emission and transmitted to the bacteriology laboratory after blinding. One hundred milligrams of faeces were suspended in 1 mL of the brain–heart infusion broth containing 30% glycerol and stored at – 80 °C. Enterobacterales were counted by plating serial dilutions of broth on Drigalski agar (bioMérieux, Marcy-l’Etoile, France).

### Determination of the ARGs repertoire and β-lactamasome

The IGC2 and the oral catalogues were annotated for the Antibiotic Resistant Determinants (ARD) using a two-step approach. First, potential ARD homologs were selected among catalogue genes using BLASTP against Mustard antibiotic resistance determinant database (http://www.mgps.eu/Mustard) [23]. Genes with ≥ 50% identity for ≥ 90% alignment coverage were selected and tested using pairwise comparative modelling (PCM), a 3-dimensional modelling-based approach [23]. This allowed the identification of a non-redundant list of 19,061 ARD from 21 families of which 5 beta-lactamase families: 627 *blaA* genes, 463 *blaB1*, 463 *blaB3*, 181 *blaC* and 89 *blaD*.

The observed richness of the ARGs repertoire, referring to genes mapping to one of the identified antibiotic-resistant determinants, was evaluated as the number of copies of genes mapping to one of the identified ARD. The relative abundance of the β-lactamasome was computed as the proportion of copies of genes mapping to any beta-lactamase family among all copies of genes mapping to one of the identified ARD.

### Determination of the β-lactamase activity

Β-lactamase activity of the faecal content was analysed in all samples (Fig. 1). For extraction of faeces, samples (stored at − 65 °C) were thawed on ice for 30 min, where after 140–380 mg of faeces material was transferred to a 2-ml Eppendorf tube by means of a spatula. Ice-cold HZn buffer (50 mM (2-hydroxyethyl)-1-piperazineethanesulfonic acid (HEPES) buffer, pH 7.5, supplemented with 50 μM ZnSO_4_) was then added to obtain samples containing 0.2 g faeces/mL. Samples were briefly mixed by means of vortexing and incubated horizontally for 1 h under mild agitation. Sample were clarified by two centrifugation steps of 15 min and 30 min (4 °C), respectively, in which the supernatant was transferred to a new 2-ml Eppendorf or finally 1.5-ml screw-cap tube.

Assays for determination of β-lactamase activity were performed in HZn buffer using 3–20 µL of freshly clarified faeces sample kept at 4 °C. Reactions were carried out in a final volume of 200 µL with 100 µM nitrocefin (Cayman Chemical Company, Ann Arbor, MI, USA). In the first assay, 10 µL of the sample was tested for the hydrolysis activity of nitrocefin. This assay was, subsequently, repeated with an adjusted sample volume if necessary. Assays were performed in 96-well microplates (SpectraPlate-96, PerkinElmer, Waltham, MA, USA) using an automated liquid handling Janus Integrator system (PerkinElmer, Waltham, MA, USA) and nitrocefin hydrolysis was monitored spectrophotometrically at a wavelength of 482 nm (EnVision microplate reader, PerkinElmer, Waltham, MA, USA). All assays always included a buffer control to assess substrate stability.

### Metagenomic analysis of the phage microbiome

The phage microbiome was analysed in samples collected at days 15, − 1, 4, 10 and 30 (Fig. 1). Phage isolation was performed using a polyethylene glycol (PEG) concentration step, as previously recommended [53]. One gram of faecal samples was weighed and homogenised in 40 mL of phosphate-buffered saline (PBS) (Sigma-Aldrich, Saint-Louis, MO, USA). The sample was then agitated with a mechanic laboratory agitator for 1 h at 4 °C, centrifuged at 17,000 × *g* for 5 min and the supernatant was filtered at 2 µm and 0.45 µm. Phages were then concentrated using PEG. One molar solid NaCl and 10% (v/v) PEG 8000 (Sigma-Aldrich, Saint-Louis, MO, USA) were dissolved into the filtered culture fluid and incubated overnight at 4 °C as recommended for a constant and stable precipitation. The solutions with the phages were pelleted by centrifugation at 5250 × *g* for 1 h at 4 °C and re-suspended in 500 µL of SM buffer (NaCl 100 mM, MgSO4.7H2O 8 mM, Tris–Cl 50 mM). Samples were treated with 10 U/ml of DNAse (Sigma-Aldrich, Saint-Louis, MO, USA) for 30 min at 37 °C followed by 10 min at 65 °C to stop the reaction. DNA was then extracted using the commercial kit “Phage DNA extraction” (Norgen Biotek Corp., Thorold, ON, Canada). DNA was purified on a sephadex column (Sigma-Aldrich, Saint-Louis, MO, USA), measured with Qubit dsDNA HS Assay kit (ThermoFisher Scientific, Waltham, MA, USA), and sequenced with the Illumina HiSeq2500 PE_250 bases method using the Kit Nextera XT with an input of 1 ng DNA. The sequence reads of the six samples of the same volunteers were pooled. They were trimmed to remove the Illumina adapters and remove low-quality reads using Atropos (v1.1.18) [54] with parameters: atropos trim -m 100 –q 20,20 –trim-n. The resulting reads were assembled using SPAdes 3.15.2 [55] with the metaviralSPAdes mode. At this step, we obtained 22 pools of contigs (1 pool per volunteer). Gene prediction was made using Prodigal (v2.6.3) [56] with –p meta option. We excluded genes lacking start and stop codons. In order to focus our analysis on contigs sufficiently large to study genetic contexts, we excluded contigs with less than 3 open reading frames (ORFs).

In order to create a non-redundant catalogue of contigs, the 22 pools of contigs were concatenated and clustered with cd-hit-est (v4.8.1) [57]. The sequence identity threshold was 0.95, the alignment must cover 90% of the shorter sequence and a sequence was clustered into the most similar cluster that meets the threshold. We used viralVerify 1.1 [55] to classify the non-redundant contigs as viral or non-viral, and only viral contig were selected for further analysis. Then, we mapped each sample read on the “viral non-redundant contigs catalog” using bowtie2 (v2.4.2 –local –very-fast-local options) [47] and exploited SAM files with samtools (v1.3.1 with the following commands: views, sort, index, idxstat) [58]. As a result, we obtained a matrix (matrix count) representing the number of reads of a sample (columns) mapping each contig reference catalog (rows) in the dataset. All the matrix counts were rarefied at 3 003 762 reads with the “rarefy” function of the vegan package in R [59].

The phage microbiome observed richness was computed as the number of phage contigs identified in each sample.

### Determination of fungal load and Candida albicans DNA concentration

The fungal loads and *Candida albicans* DNA concentration were analysed in all available samples. For each faecal sample, 250 mg were processed using the repeated bead beating plus column protocol described elsewhere [60] (Fig. 1). A FastPrep-24™ device (MP Biomedicals, Santa Ana, CA, USA) was used instead of a Mini-BeadbeaterTM. Faecal DNA levels were quantified with the Qubit dsDNA Broad Range Kit (Invitrogen, Waltham, MA, USA), and only samples with a concentration above 50 ng/μL were considered in the analysis.

A TaqMan qPCR protocol, using a double dye MGB 5′ 6-FAM-labelled probe (Eurogentec, Seraing, Belgium), with the following conditions: 2 min at 50 °C, 10 min at 95 °C, 15 s at 95 °C and 1 min at 65 °C, the last two steps repeated for 45 cycles, was used to measure fungal DNA levels [61]. Samples were processed in two sets of duplicates, in two independent rounds. The fungal load was estimated for each sample as the ratio of the fungal DNA levels to the faecal DNA levels [62].

A TaqMan qPCR protocol in the following conditions: 2 min at 50 °C, 10 min at 95 °C, 15 s at 95 °C and 1 min at 62 °C, the last two steps repeated for 45 cycles, was used to quantify *C. albicans* DNA levels. 7.5 μL of the extracted faecal DNA, at a 1:10 dilution, were used as a template, using probes and primers described by Guiver et al. 2001, at 0.1 μM and 0.2 μM, respectively [63]. Samples were processed in two sets of duplicates, in two independent rounds.

The presence of qPCR inhibitors in the samples was verified in all samples, diluted at 1:10, using the Universal Exogenous qPCR Positive Control for TaqMan® Assay (Eurogentec, Seraing, Belgium), with a Cy®5-QXL®670 Probe system (Eurogentec, Seraing, Belgium). Manufacturer’s recommendations were followed, using the target Ct > 30 option.

### Targeted-metagenomic analysis of the fungal microbiome

All samples were processed to study the fungal microbiota (Fig. 1). The Internal Transcriber Spacers (ITS) 1 region was targeted for the preparation of the amplicon libraries. The amplicons were produced by PCR using the ITS1F and ITS2 primers in the following conditions [64, 65]: 95 °C for 3 min, 25 cycles of 95 °C for 30 s, 55 °C for 30 s and 72 °C for 30 s, 72 °C for 5 min and cooling at 4 °C, and their size were verified with a Bioanalyzer DNA 1000 chip (Agilent Technologies, Santa Clara, CA, USA). The purification of the amplicon was performed with AMPure XP (Beckman Coulter, Brea, CA, USA) as described in the 16S Metagenomic Sequencing Library Preparation guide [66]. The adapters were attached with the Nextera XT Index Kit (Illumina, San Diego, CA, USA) and index PCRs were done in the following conditions: 95 °C for 3 min, 8 cycles of 95 °C for 30 s, 55 °C for 30 s and 72 °C for 30 s, 72 °C for 5 min and cooling at 4 °C. AMPure XP (Beckman Coulter, Brea, CA, USA) was used to purify the PCR products and a Bioanalyzer DNA 1000 chip allowed their verification and their quantification. Samples concentrations were normalised at 4 nM and 5 µL of each diluted sample was pooled into a library and a PhiX sequencing control was prepared according to the manufacturer’s guidelines. Libraries were sequenced on Illumina MiSeq platform (Illumina, San Diego, CA, USA) with the MiSeq Reagent Kit V3 in 300 bp paired-end.

The sequencing allowed the recovery of 8,819,635 amplicons from the ITS1 region. The SHAMAN pipeline was used to remove the singletons and chimera amplicons, resulting in a total of 56,634 amplicons [67]. The remaining amplicons were clustered in 4648 OTUs using a cut-off value of 97% identity. Five hundred fifty-one OTUs could be associated with fungal sequences using the Unite database and on these OTUs, 340 were present in at least two faecal samples and were kept for further analysis. A first round of annotation was performed on SHAMAN against the UNITE database (rev. 8.0) and then a second round was performed against a more recent release of UNITE (rev. 8.2). The OTUs that could not be annotated after these two rounds submitted to a classic BLASTN and only hits matched with a similarity above 97% to reference genomes were conserved. The abundances and weighted non-null normalised counts tables were generated with SHAMAN [67].

The observed richness of the fungal microbiome was computed as the number of unique fungal OTUs identified in each sample.

### Non-targeted analysis of the metabolome

The metabolome was analysed in all collected samples (Fig. 1). Experimental methods and parameters for the non-targeted approach were carried out by liquid chromatography and high-resolution mass spectrometry (LC-HRMS) as detailed in [68, 69]. Briefly, eight volumes of frozen acetonitrile (− 20 °C) containing internal standards (labelled IS mix of amino acids at 10 µg/mL) were added to 100 µL serum samples and vortexed. The resulting samples were then sonicated for 10 min and centrifuged for 2 min at 10,000 × *g* at 4 °C. Supernatants were incubated at 4 °C for 1 h for a slow protein precipitation process. Samples were centrifuged for 20 min at 20,000 g at 4 °C. Supernatants were transferred to another series of tubes and then dried and stored at – 80 °C before LC-HRMS analyses. Pellets were diluted 3-fold and reconstituted with H2O/ACN (20/80).

Non-targeted approach experiments were performed using a HILIC phase chromatographic column, ZIC-pHILIC 5 µm, 2.1 × 150 mm at 15 °C (Merck, Darmstadt, Germany), and on a UPLC Waters Acquity (Waters, Milford, MA, USA) coupled to Q-Exactive mass spectrometer (Thermo Fisher Scientific, Waltham, MA, USA). Processing steps were carried out using the R software [70]. Peak detection, correction, alignment and integration were processed using XCMS R package with CentWave algorithm [71, 72] and workflow4metabolomics platforms [73]. The resulted datasets were log10 normalised, filtered and cleaned based on quality control (QC) samples [74]. The features were then putatively annotated based on their mass over charge ratio (m/z) as well as retention time using a local database as described previously [75] and then validated based on MS/MS experiments. The remaining features were either characterised using public repositories [76, 77] or discarded when feature status is still unknown to remove noise and artifact signals. The relative abundance of all annotated chemical features was then summed and computed as a total signal, named ‘total useful signal’, for each sample. The observed richness of the metabolome was computed as the number of unique chemical species identified in each sample.

### Analysis of the cholesterol conversion rate into coprostanol

The microbiota-dependent catabolism of the cholesterol in faeces was analysed in all collected samples (Fig. 1). Sterols and stanols were extracted from faeces as follows. Faeces were weighted (~ 50 mg) and resuspended in 1% formic acid to a final concentration of 167 µg/µL. The mixture was homogenised using a Precellys Evolution instrument (Bertin Instruments, Montigny-le-Bretonneux, France) using the ‘soft program’. Volume equivalent to 1 mg of dried faeces was supplemented with deuterated internal standards (cholesterol d7 and coprostanol d5) and sterols and stanols extracted with 1.2 mL of methanol/chloroform (2:1 v/v) and 320 µL deionised water. Phase separation was triggered with 400 µL chloroform and 400 µL water. The mixture was centrifuged for 10 min at 3600 × *g* and the lower phase was collected and dried. Sterols and stanols were derivatised for compatibility with GC–MS analysis using 60 µL of BSTFA (with 1% TMCS). The solution was heated at 80 °C for 1 h, dried and resuspended in 0.1% BSTFA in cyclohexane before injection in the GC–MS. Samples were injected at 250 °C in split mode and sterols/stanols were separated on a 50 m × 0.25 mm, 0.25 µm DB-5MS column. Sterols and stanols were ionised using electronic impact (EI) and analysed in SIM mode using m/z 136 for squalen, m/z 393 for lanosterol, m/z 366 for desmosterol, m/z 443 for lathosterol, m/z 329 for cholesterol, m/z 382 for campesterol, m/z 394 for stigmasterol, m/z 381 for β-sitosterol, m/z 370 for coprostanol and m/z 398 for ethylcoprostanol as quantitative ions. The ability of the gut microbiota to convert cholesterol into its major reduced form (i.e. coprostanol) was calculated as the ratio of the faecal coprostanol concentration to the sum of the faecal coprostanol and cholesterol concentrations, hence the use of the generic term “cholesterol conversion rate” throughout the manuscript.

### Analysis of the biliary acid transformation

The metabolism of the biliary acids in faeces was analysed in all collected samples (Fig. 1). All chemicals and solvents were of the highest purity available. Cholic acid (CA), deoxycholic acid (DCA), chenodeoxycholic acid (CDCA), ursodeoxycholic acid (UDCA), lithocholic acid (LCA), hyocholic acid HCA, hyodeoxicholic acid (HDCA), glyco and tauro derivatives were obtained from Sigma-Aldrich (Saint Quentin Fallavier, France). 3α-sulfate derivatives were a generous gift of J. Goto (Niigita University of Pharmacy and Applied Life Science, Niigata, Japan) and 7α-cholic acid (CA-7S) was from Cayman Chemical (Ann Arbor, MI, USA). 23-NOR-5β-cholanoic acid-3α,12α diol, all muricholic acids, glyco, tauro derivatives and iso, keto bile acids were purchased from Steraloids Inc (Newport, RI, USA). Acetic acid, ammonium carbonate, ammonium acetate and methanol were of HPLC grade and purchased from Sigma-Aldrich (Saint Quentin Fallavier, France).

Bile acid molecular species concentrations were measured by HPLC coupled to tandem mass spectrometry (HPLC–MS/MS) as previously described with slight modification [78]. Two microlitres of an internal standard solution (23-nor-5β-cholanoic acid-3α, 12α-diol at 1 mg/ml) was added to 10–50 mg of faeces lyophilised samples using a Lyovapor L200 (Buchi, Villebon-sur-Yvette, France). For 15–20 mg lyophilised faeces samples, 2 ml of NaOH (0.1 M) was added and incubated for 1 h at 60 °C before adding 4 ml of water. The solution was homogenised by two 10 s runs in an Ultra-Turrax disperser (IMLAB, Lille, France). The preanalysis cleanup procedure was achieved by centrifugation (12,000 × *g* for 20 min) followed by solid-phase extraction using reversed-phase silica cartridges (HLB Oasis, Waters, Milford, MA, USA), and we used a 5500Q-trap (AB Sciex, Framingham, MA, USA) as mass spectrometer.

The hydrophobicity index reflects BA hydrophobicity, taking into account the concentration and the retention time of different BAs on a C18 column with a methanol gradient; lithocholic acid has the highest retention time, tauroursodeoxycholic acid-3S has the lowest.

The ability of the gut microbiota to metabolise the biliary acids was computed as the ratio of the secondary biliary acids (LCA and DCA) to the total concentration of the faecal content in biliary acids.

### Data analysis

Data were classified between high dimensional variables relative to the structure of the bacterial, phage and fungal microbiomes, and metabolome, and low dimensional variables (observed richness of the bacterial, phage and fungal microbiomes, observed richness of the ARGs repertoire and metabolome, relative abundance of the β-lactamasome, total bacterial counts, β-lactamase activity, fungal load and *Candida albicans* DNA levels, cholesterol conversion rate into coprostanol, and biliary acid transformation).

Baseline was defined at day 0, and the baseline sample was defined as the sample obtained at day − 1. If this sample at day − 1 was not available, the sample obtained at day − 7 was considered as the baseline, or the one obtained at day − 15 if this latter was also missing.

For high dimensional variables, we computed for each subject the Spearman’s correlation coefficient (s) of the structure of the studied system between baseline and each sampling day. These correlation coefficients were used to evaluate the change from baseline of the structure of the system. Among low dimensional variables, all variables, except those relative to the observed richness of the systems, were log10 transformed before analysis, and we computed the change from baseline at each sampling time as the difference of the values at each time.

In order to study the variability between subjects and within subjects for each variable before the administration of antibiotics, we analysed the low dimensional variables using a linear mixed effect model (lmer function of R package lme4), treating subjects as random effects. We respectively estimated the between within-subjects variabilities as the coefficient of variation of the random effect and of the residual error estimated in the model.

In order to study the perturbation of systems, we computed a raw distance from the baseline, that increases with the extent of the perturbation of each system, regardless of the direction of the perturbation. It was calculated at each sampling time as 1-s^2^ for high-dimensional variables (with s being the Spearman’s correlation coefficient as described above) and as the absolute change from baseline for low dimensional variables.

Raw distances from the baseline were normalised to address the effect of intra-individual variations of the systems before the start of antibiotic treatment. Normalisation was made for each subject by dividing distances from baseline by the individual average of the distances from baseline computed before the beginning of antibiotic treatment (at days − 7 and − 15). In the case of missing samples at days − 7 and − 15, the normalisation was used as the median of the average raw distances computed for all other subjects.

We studied the effect of antibiotics on the gut content using both fixed endpoints (days 4, 7, 10, 30 and 90) and areas under the curve between baseline and days 10 (AUC_D0-D10_) and 30 (AUC_D0-D30_). Metrics used were the changes from baseline for low dimensional variables and the normalised distances for high dimensional variables. AUCs were computed using the trapezoidal rule, using the actual date and time of stool emission. AUCs were standardised using the observed delay between baseline and the actual time of collection of the day 10 (for AUC_D0-D10_) or day 30 (for AUC_D0-D30_) sample. We used the non-parametric Wilcoxon test to compare these metrics at fixed sampling times, the AUC_D0-D10_, and the AUC_D0-D30_ to 0 for low dimensional variables, and to 1, 10 or 30 for high dimensional variables and their AUC_D0-D10_ AUC_D0-D30_, respectively. We also compared the effect of the two antibiotics on the microbiota using the non-parametric Wilcoxon test.

Next, we defined for each subject and system the maximal perturbation as the maximal normalised distance from baseline observed between the baseline and day 10, and maximal resilience as the minimal normalised distance from baseline observed on days 15, 30 or 90. Pairwise relations between the level of maximal perturbation for each system were investigated using Spearman’s correlation coefficients and comparing them to 0. A similar analysis was performed to study the relationship between the maximal resilience of systems.

Finally, the relationship between the composition of the microbiome at baseline (studied using the low dimensional variables) and the maximal perturbation and resilience of studied systems was assessed using Spearman’s correlation coefficient and its test to 0.

All statistical tests were bilateral, with a type-I error fixed to 0.05. As the present work constitutes an exploratory analysis, *P* values were not corrected for multiple testing. In order to assess the robustness of the analysis, Benjamini and Hochberg correction of *p*-values was performed as a sensitivity analysis to correct for the false discovery rate. The correction was made globally for each analysis.

### Supplementary Information

**Additional file 1: Supplementary Figure S1.** Evolution of the gut microbiota and stool components in the 2 healthy volunteers with detectable levels of antibiotics in faeces. **Supplementary Table S1.** Effect of the antibiotics on each of the studied components, according to the treatment group in the 22 healthy volunteers included in the CEREMI trial. **Supplementary Table S2.** Comparison of ceftriaxone and cefotaxime effect on each of the studied component, according to the treatment group in the 22 healthy volunteers included in the CEREMI trial. **Supplementary Table S3.** Spearman’s correlations of the maximal perturbations of studied gut microbiota and stool components in the 22 healthy volunteers included in the CEREMI trial. **Supplementary Table S4.** Spearman’s correlations of the maximal resiliences of studied gut microbiota and stool components in the 22 healthy volunteers included in the CEREMI trial. **Supplementary Table S5.** Spearman’s correlations between the baseline characteristics and the maximal perturbations of the studied gut microbiota and gut components in the 22 healthy volunteers included in the CEREMI trial. **Supplementary Table S6.** Spearman’s correlations between the baseline characteristics and the maximal resiliences of the studied gut microbiota and gut components in the 22 healthy volunteers included in the CEREMI trial. **Supplementary Table S7.** Baseline characteristics, maximal perturbations and maximal resiliences of the studied gut microbiota and gut components in the 22 healthy volunteers included in the CEREMI trial.

## Data Availability

The metagenomic shotgun sequencing data of bacterial microbiome are available from the European Nucleotide Archive under accession number PRJEB58157 (www.ebi.ac.uk/ena/browser/view/PRJEB58157). The metagenomic shotgun sequencing data of phage microbiome are available from the European Nucleotide Archive under accession number PRJEB58815 (www.ebi.ac.uk/ena/browser/view/PRJEB58815). The metagenomic shotgun sequencing data of fungal microbiome are available from the European Nucleotide Archive under accession number PRJNA956890 (www.ncbi.nlm.nih.gov/bioproject/956890). The metabolomics data are available from the Metabolights repository under accession number MTBLS6771 (www.ebi.ac.uk/metabolights/MTBLS6771). The clinical data will be made available upon request to the corresponding author.
