# Supplementary material for: Perturbation and resilience of the gut microbiome up to 3 months after β-lactams exposure in healthy volunteers suggest an important role of microbial β-lactamases
Source: Microbiome. 2024 Mar 12;12:50. doi: 10.1186/s40168-023-01746-0 (PMC10929231; doi:10.1186/s40168-023-01746-0)
Supplement: Supplementary file 2 — Additional file 1: Supplementary Figure S1. Evolution of the gut microbiota and stool components in the 2 healthy volunteers with detectable levels of antibiotics in faeces. Supplementary Table S1. Effect of the antibiotics on each of the studied components, according to the treatment group in the 22 healthy volunteers included in the CEREMI trial. Supplementary Table S2. Comparison of ceftriaxone and cefotaxime effect on each of the studied component, according to the treatment group in the 22 healthy volunteers included in the CEREMI trial. Supplementary Table S3. Spearman’s correlations of the maximal perturbations of studied gut microbiota and stool components in the 22 healthy volunteers included in the CEREMI trial. Supplementary Table S4. Spearman’s correlations of the maximal resiliences of studied gut microbiota and stool components in the 22 healthy volunteers included in the CEREMI trial. Supplementary Table S5. Spearman’s correlations between the baseline characteristics and the maximal perturbations of the studied gut microbiota and gut components in the 22 healthy volunteers included in the CEREMI trial. Supplementary Table S6. Spearman’s correlations between the baseline characteristics and the maximal resiliences of the studied gut microbiota and gut components in the 22 healthy volunteers included in the CEREMI trial. Supplementary Table S7. Baseline characteristics, maximal perturbations and maximal resiliences of the studied gut microbiota and gut components in the 22 healthy volunteers included in the CEREMI trial. [file 40168_2023_1746_MOESM1_ESM.pdf]

Supplementary Appendix

Supplementary Figure S1. Evolution of the gut microbiota and stool components in the 2 healthy volunteers with detectable levels of antibiotics in faeces.

For 'low dimensionality' systems, the log10 fold changes from baseline are presented (except for variables relative to system's richness which were not transformed), whereas for 'high-dimensionality' systems we depicted the normalized distance from baseline. In each graph the x-axis indicates the time following antibiotic administration, whereas the y-axis corresponds to the change from baseline (positive and/or negative) with the unit in brackets. The two selected individual are represented by a blue and red line, respectively. The dotted lines correspond to the median of the remaining 20 subjects. Cholesterol conv. rate, Cholesterol conversion rate; BA, Bile acids.

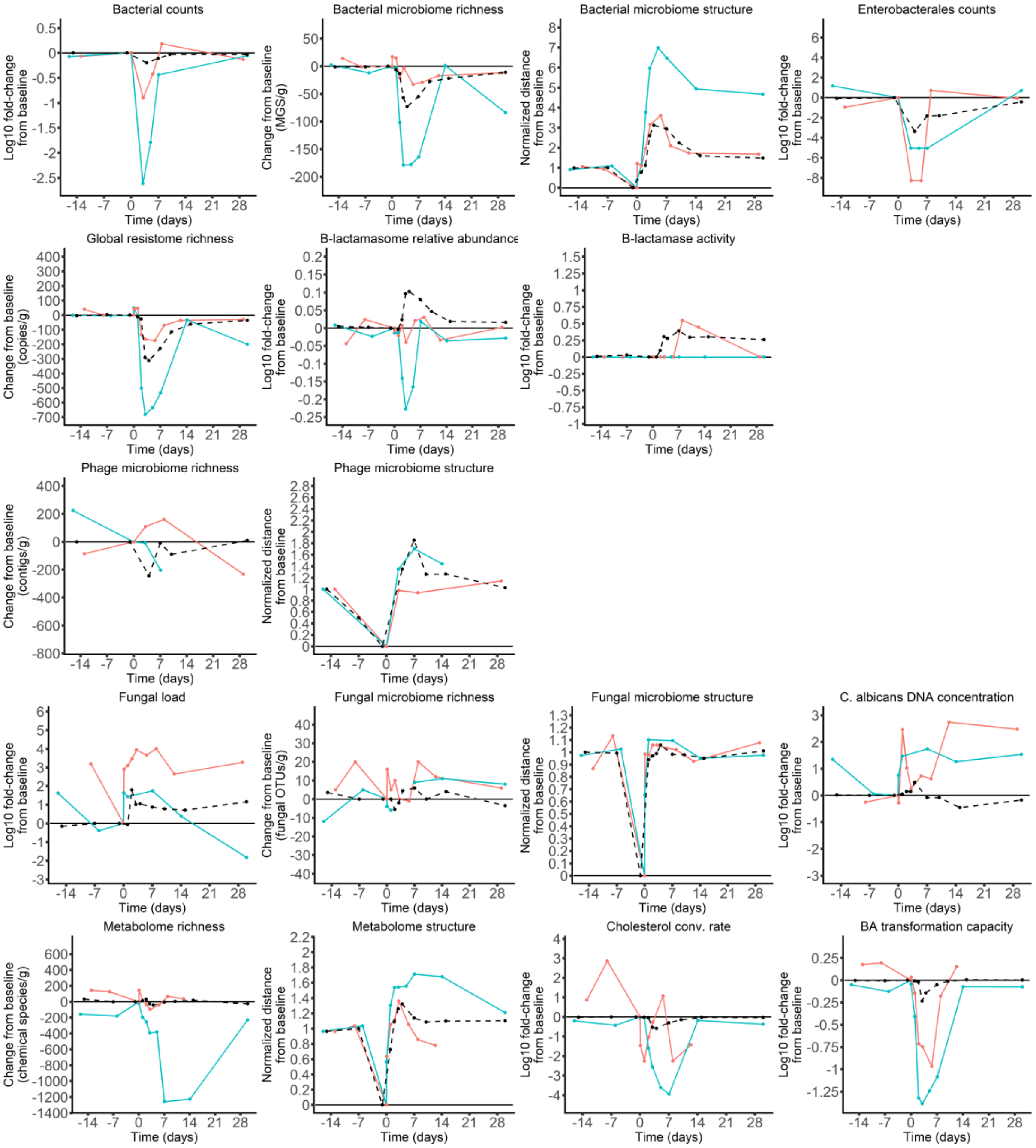

Supplementary Table S1. Effect of the antibiotics on each of the studied components, according to the treatment group in the 22 healthy volunteers included in the CEREMI trial.
For 'low dimensionality' systems, the log10 fold changes from baseline were used (except for variables relative to system's richness which were not transformed), whereas for 'high-dimensionality' systems we used the normalized distance from baseline.
Baseline data were obtained from the day -1 sample. AUC D0D10 (respectively AUC D0D30) were normalized by the actual time observed between baseline and day 10 (respectively day 30). Data are presented as median (min; max). P-values refer to the result of non-parametric Wilcoxon test. Q-value corresponds to the test after Benjamini & Hochberg's correction. P-values and Q-values in bold represent values below 0.05.

Endpoint	n	med (min; max)	P-value	Q-value
**Bacterial counts (log10 CFU/g)**				
AUC D0D10	22	-1.2 (-15; 2.3)	**7.4E-03**	**1.3E-02**
AUC D0D30	21	-2.9 (-16.3; 8.5)	8.2E-02	1.2E-01
Day 4	22	-0.3 (-2.6; 0.2)	**8.1E-04**	**1.8E-03**
Day 7	22	-0.1 (-1.8; 0.3)	1.4E-01	2.0E-01
Day 10	22	0 (-0.7; 0.3)	2.2E-01	2.8E-01
Day 30	21	-0.1 (-0.3; 0.5)	4.5E-01	5.3E-01
Day 90	-	-	-	-
**Bacterial microbiome richness (MGS/g)**				
AUC D0D10	22	-411 (-1460; -76.2)	**4.8E-07**	**7.3E-06**
AUC D0D30	20	-783 (-2590; 45.6)	**3.8E-06**	**1.6E-05**
Day 4	22	-73 (-179; -3)	**4.8E-07**	**7.3E-06**
Day 7	22	-55 (-178; 21)	**8.0E-05**	**2.4E-04**
Day 10	22	-28.5 (-164; 5)	**6.1E-05**	**1.9E-04**
Day 30	21	-11.5 (-84; 21)	**2.7E-03**	**5.5E-03**
Day 90	22	-13 (-74; 35)	**1.9E-02**	**3.0E-02**
**Bacterial microbiome structure**				
AUC D0D10	22	23.7 (13; 49.1)	**4.8E-07**	**4.6E-06**
AUC D0D30	20	55.7 (36.9; 151)	**1.9E-06**	**1.4E-05**
Day 4	22	3.1 (1.1; 7.7)	**4.8E-07**	**4.6E-06**
Day 7	22	3 (1.6; 7)	**4.8E-07**	**4.6E-06**
Day 10	22	2.2 (1.3; 6.5)	**4.8E-07**	**4.6E-06**
Day 30	20	1.5 (0.9; 4.7)	**3.6E-05**	**1.5E-04**
Day 90	21	1.7 (0.9; 4.6)	**4.8E-06**	**2.7E-05**
**Enterobacterales counts (log10 CFU/g)**				
AUC D0D10	22	-22.1 (-57.5; 24.1)	**4.2E-05**	**1.5E-04**
AUC D0D30	21	-48.8 (-163; 145)	**4.3E-04**	**1.1E-03**
Day 4	22	-3.5 (-8.3; 0)	**6.0E-05**	**1.9E-04**
Day 7	22	-2.2 (-9; 5.9)	**5.9E-04**	**1.4E-03**
Day 10	22	-1.8 (-9; 4.9)	**6.9E-04**	**1.6E-03**
Day 30	21	-0.3 (-2.6; 6.9)	3.3E-01	4.1E-01
Day 90	22	0.1 (-3; 3.5)	8.5E-01	8.8E-01
**Global resistome richness (copies/g)**				
AUC D0D10	22	-1850 (-4690; 116)	**9.5E-07**	**7.3E-06**
AUC D0D30	20	-3510 (-9320; 1000)	**5.7E-06**	**2.3E-05**
Day 4	22	-312 (-682; -122)	**4.0E-05**	**1.5E-04**
Day 7	22	-229 (-635; 176)	**1.2E-05**	**4.6E-05**

Endpoint	n	med (min; max)	P-value	Q-value
Day 10	22	-114 (-534; 111)	**2.1E-05**	**7.9E-05**
Day 30	20	-37.5 (-283; 173)	**3.3E-02**	5.2E-02
Day 90	21	-51 (-320; 217)	1.2E-01	1.7E-01
**β-lactamasome relative abundance (log10)**				
AUC D0D10	22	0.6 (-1.2; 1.4)	**2.1E-04**	**5.7E-04**
AUC D0D30	20	1 (-1.4; 2.8)	**1.4E-03**	**3.0E-03**
Day 4	22	0.1 (-0.2; 0.2)	**5.0E-04**	**1.3E-03**
Day 7	22	0.1 (-0.2; 0.2)	**2.6E-04**	**6.9E-04**
Day 10	22	0 (-0.1; 0.1)	**6.5E-05**	**2.0E-04**
Day 30	20	0 (0; 0.1)	1.6E-01	2.2E-01
Day 90	21	0 (-0.1; 0.1)	1.9E-01	2.5E-01
**β-lactamase activity (log10 nmol/min.g)**				
AUC D0D10	22	2.4 (-2.2; 8.7)	**8.0E-04**	**1.8E-03**
AUC D0D30	21	6.8 (-6.5; 26.7)	**5.9E-04**	**1.4E-03**
Day 4	22	0.2 (-0.6; 1.4)	**4.4E-02**	6.6E-02
Day 7	22	0.4 (-0.9; 1.2)	**1.9E-03**	**3.9E-03**
Day 10	22	0.3 (-0.2; 1.5)	**2.1E-04**	**5.7E-04**
Day 30	21	0.2 (-0.4; 0.8)	**2.9E-03**	**5.8E-03**
Day 90	22	0.1 (-0.4; 1)	**4.4E-02**	6.6E-02
**Phage microbiome richness (contigs/g)**				
AUC D0D10	20	-1330 (-4190; 2230)	**8.3E-03**	**1.4E-02**
AUC D0D30	14	-2030 (-5670; 8050)	1.5E-01	2.1E-01
Day 4	19	-227 (-708; 219)	**3.9E-03**	**7.7E-03**
Day 7	-	-	**-**	**-**
Day 10	20	-90 (-421; 371)	6.4E-02	9.5E-02
Day 30	14	-3.5 (-233; 206)	>9.9E-01	>9.9E-01
Day 90	-	-	-	-
**Phage microbiome structure**				
AUC D0D10	20	12.7 (9.7; 19.8)	**5.7E-06**	**3.1E-05**
AUC D0D30	14	32 (27.4; 47)	**4.0E-03**	**8.7E-03**
Day 4	19	1.4 (0.9; 2.6)	**7.2E-05**	**2.6E-04**
Day 7	-	-	**-**	**-**
Day 10	20	1.3 (0.9; 1.9)	**1.9E-05**	**8.0E-05**
Day 30	14	1 (0.7; 1.3)	7.1E-01	7.6E-01
Day 90	-	-	-	-
**Fungal load (log10)**				
AUC D0D10	20	6.7 (-9.1; 47.5)	**8.3E-03**	**1.4E-02**
AUC D0D30	19	35 (-39.2; 114)	**5.3E-03**	**9.8E-03**
Day 4	14	1.3 (-0.7; 5.4)	**4.2E-02**	6.5E-02
Day 7	19	0.9 (-0.6; 5.7)	**9.7E-04**	**2.1E-03**
Day 10	20	1.1 (-2.3; 4)	**6.4E-03**	**1.1E-02**
Day 30	19	1.2 (-1.8; 4.4)	**1.1E-02**	**1.8E-02**
Day 90	20	0.6 (-3.6; 6.8)	1.8E-01	2.4E-01
**Fungal microbiome richness (fungal OTUs/g)**				
AUC D0D10	21	20 (-250; 181)	5.2E-01	6.0E-01

**Endpoint**	**n**	**med (min; max)**	P-value	Q-value
AUC D0D30	20	52.1 (-800; 520)	3.7E-01	4.5E-01
Day 4	22	2 (-27; 24)	6.4E-01	7.1E-01
Day 7	22	6 (-40; 33)	2.0E-01	2.6E-01
Day 10	22	4 (-20; 21)	3.1E-01	3.9E-01
Day 30	21	-2.5 (-32; 32)	5.5E-01	6.3E-01
Day 90	22	0 (-36; 28)	4.7E-01	5.5E-01
**Fungal microbiome structure**				
AUC D0D10	21	9.9 (9; 12)	>9.9E-01	>9.9E-01
AUC D0D30	20	29.4 (24.6; 35.1)	6.7E-01	7.3E-01
Day 4	15	1.1 (0.8; 1.1)	2.5E-01	3.1E-01
Day 7	21	1 (0.8; 1.3)	9.2E-01	9.5E-01
Day 10	21	1 (0.8; 1.3)	4.3E-01	5.0E-01
Day 30	20	1 (0.7; 1.2)	5.2E-01	5.9E-01
Day 90	20	1 (0.7; 1.3)	5.7E-01	6.3E-01
**C. albicans DNA concentration (log10)**				
AUC D0D10	20	-0.4 (-22; 15.3)	7.8E-01	8.2E-01
AUC D0D30	19	-7.2 (-58.7; 59.1)	7.1E-01	7.6E-01
Day 4	14	0.4 (-1.6; 2.4)	6.3E-01	7.0E-01
Day 7	19	0 (-2.7; 1.8)	6.5E-01	7.1E-01
Day 10	20	0 (-2.8; 1.7)	3.9E-01	4.7E-01
Day 30	19	0 (-2; 2.5)	8.1E-01	8.5E-01
Day 90	20	-0.1 (-2.8; 0.8)	1.8E-01	2.4E-01
**Metabolome richness (chemical species/g)**				
AUC D0D10	21	-162 (-4400; 5720)	2.6E-01	3.3E-01
AUC D0D30	19	-435 (-23400; 18400)	3.1E-01	3.9E-01
Day 4	22	-61 (-394; 693)	**1.2E-02**	**2.0E-02**
Day 7	22	-19.5 (-380; 515)	6.2E-01	7.0E-01
Day 10	22	4 (-1260; 566)	9.9E-01	>9.9E-01
Day 30	21	-27 (-229; 631)	8.7E-02	1.3E-01
Day 90	22	-28 (-97; 520)	4.5E-01	5.3E-01
**Metabolome structure**				
AUC D0D10	21	11.5 (9; 17.9)	**4.10E-05**	**1.6E-04**
AUC D0D30	19	33.8 (27.3; 45.2)	**6.40E-04**	**1.6E-03**
Day 4	22	1.3 (0.9; 2.1)	**2.40E-06**	**1.6E-05**
Day 7	20	1.1 (0.9; 1.7)	**1.20E-03**	**2.9E-03**
Day 10	21	1.1 (0.7; 2.1)	8.90E-02	1.2E-01
Day 30	19	1.1 (0.8; 1.6)	5.50E-02	8.0E-02
Day 90	21	1.2 (0.8; 1.8)	**2.20E-02**	**3.7E-02**
**Cholesterol conversion rate (log10)**				
AUC D0D10	21	-4.8 (-24.4; 8.7)	**1.0E-03**	**2.1E-03**
AUC D0D30	19	-9.3 (-56.2; 17.9)	**9.7E-04**	**2.1E-03**
Day 4	22	-0.6 (-3.3; 3.5)	**4.2E-03**	**8.1E-03**
Day 7	20	-0.3 (-3.6; 1.1)	**6.4E-03**	**1.1E-02**
Day 10	21	-0.2 (-4.3; 2.6)	**4.9E-03**	**9.2E-03**
Day 30	19	0 (-1; 0.2)	1.0E-01	1.4E-01

**Endpoint**	**n**	**med (min; max)**	P-value	Q-value
Day 90	21	0 (-1.8; 1.8)	6.8E-01	7.4E-01
**Bile acid transformation capacity (log10)**				
AUC D0D10	21	-0.9 (-10.5; 0.7)	**5.2E-05**	**1.8E-04**
AUC D0D30	19	-1 (-12.7; 3.1)	**7.1E-03**	**1.2E-02**
Day 4	21	-0.2 (-1.4; 0)	**2.9E-06**	**1.4E-05**
Day 7	21	-0.1 (-1.2; 0.1)	**4.3E-03**	**8.2E-03**
Day 10	21	0 (-1.1; 0.1)	**1.9E-02**	**3.0E-02**
Day 30	19	0 (-0.1; 0.4)	7.4E-01	7.9E-01
Day 90	20	0 (-0.4; 0.4)	>9.9E-01	>9.9E-01

Supplementary Table S2. Comparison of ceftriaxone and cefotaxime effect on each of the studied component, according to the treatment group in the 22 healthy volunteers included in the CEREMI trial.

For 'low dimensionality' systems, the log10 fold changes from baseline were used (except for variables relative to system's richness which were not transformed), whereas for 'high-dimensionality' systems we used the normalized distance from baseline.

Baseline data were obtained from the day -1 sample. AUC D0D10 (respectively AUCD0D30) were normalized by the actual time observed between baseline and day 10 (respectively day 30). Data are presented as median (min; max). P-values refer to the result of non-parametric Wilcoxon test. Q-value corresponds to the test after Benjamini & Hochberg's correction. P-values and Q-values in bold represent values below 0.05.

Endpoint	Ceftriaxone		Cefotaxime		P-value	Q-value
	n	med (min; max)	n	med (min; max)		
**Bacterial counts (log10 CFU/g)**						
AUC D0D10	11	-0.7 (-15; 2.3)	11	-1.7 (-4.2; 1.6)	7.0E-01	9.6E-01
AUC D0D30	10	-0.9 (-16.3; 8.5)	11	-3 (-9.6; 7.4)	>9.9E-01	>9.9E-01
Day 4	11	-0.1 (-2.6; 0.2)	11	-0.3 (-0.9; 0.1)	5.2E-01	9.4E-01
Day 7	11	-0.1 (-1.8; 0.3)	11	-0.2 (-0.7; 0.3)	6.1E-01	9.4E-01
Day 10	11	-0.1 (-0.7; 0.3)	11	0 (-0.4; 0.3)	2.2E-01	9.4E-01
Day 30	10	0 (-0.3; 0.5)	11	-0.1 (-0.3; 0.4)	2.0E-01	9.4E-01
Day 90	-	-	-	-	-	-
**Bacterial microbiome richness (MGS/g)**						
AUC D0D10	11	-452 (-1270; -76.2)	11	-403 (-1460; -148)	6.5E-01	9.4E-01
AUC D0D30	10	-855 (-2230; 45.6)	10	-736 (-2590; -476)	>9.9E-01	>9.9E-01
Day 4	11	-64 (-179; -11)	11	-81 (-170; -3)	4.4E-01	9.4E-01
Day 7	11	-56 (-178; 1)	11	-54 (-118; 21)	4.1E-01	9.4E-01
Day 10	11	-47 (-164; 1)	11	-24 (-78; 5)	8.2E-02	9.4E-01
Day 30	10	-13.5 (-84; 3)	11	-11.5 (-76; 21)	9.4E-01	>9.9E-01
Day 90	11	-13 (-57; 18)	11	-15 (-74; 35)	9.4E-01	>9.9E-01
**Bacterial microbiome structure**						
AUC D0D10	11	18.7 (13; 49.1)	11	24.9 (14; 48.9)	4.0E-01	9.9E-01
AUC D0D30	10	57.4 (39.2; 151)	10	52.9 (36.9; 109)	6.3E-01	9.9E-01
Day 4	11	2 (1.1; 6)	11	3.2 (1.4; 7.7)	2.4E-01	9.9E-01
Day 7	11	2.9 (1.6; 7)	11	3 (1.6; 6.6)	8.5E-01	9.9E-01
Day 10	11	2.7 (1.3; 6.5)	11	2.1 (1.3; 3.3)	2.2E-01	9.9E-01
Day 30	10	1.7 (0.9; 4.7)	10	1.4 (1; 2.4)	6.8E-01	9.9E-01
Day 90	11	1.7 (0.9; 4.6)	10	1.6 (1; 3.3)	6.5E-01	9.9E-01
**Enterobacterales counts (log10 CFU/g)**						
AUC D0D10	11	-22.2 (-57.5; -15.7)	11	-14.5 (-53.2; 24.1)	7.6E-02	9.4E-01
AUC D0D30	10	-52.6 (-163; -19.8)	11	-34.8 (-79.5; 145)	2.8E-01	9.4E-01
Day 4	11	-3.5 (-7.2; -1.3)	11	-3.4 (-8.3; 0)	7.5E-01	9.6E-01
Day 7	11	-3.6 (-9; 1.1)	11	-0.8 (-8.3; 5.9)	8.8E-02	9.4E-01
Day 10	11	-2.6 (-9; 0)	11	-1.3 (-3.7; 4.9)	5.6E-02	9.4E-01
Day 30	10	0.2 (-1.8; 1.7)	11	-0.8 (-2.6; 6.9)	1.5E-01	9.4E-01
Day 90	11	0.3 (-0.8; 2.3)	11	-0.2 (-3; 3.5)	4.0E-01	9.4E-01
**Global resistome richness (copies/g)**						
AUC D0D10	11	-1790 (-4690; -522)	11	-1910 (-2900; 116)	8.5E-01	9.6E-01

AUC D0D30	10	-3680 (-9320; 1000)	10	-3190 (-6900; -20.2)	4.8E-01	9.4E-01
Day 4	11	-285 (-682; -122)	11	-319 (-476; -166)	5.3E-01	9.4E-01
Day 7	11	-294 (-635; -56)	11	-212 (-374; 176)	3.3E-01	9.4E-01
Day 10	11	-168 (-534; -13)	11	-78 (-175; 111)	7.6E-02	9.4E-01
Day 30	10	-69.5 (-200; 67)	10	-27 (-283; 173)	7.4E-01	9.6E-01
Day 90	11	-46 (-250; 217)	10	-57 (-320; 209)	8.3E-01	9.6E-01
**β-lactamasome relative abundance (log10)**						
AUC D0D10	11	0.6 (-1.2; 1.4)	11	0.7 (0; 1.2)	6.5E-01	9.4E-01
AUC D0D30	10	1.1 (-1.4; 2.8)	10	1 (-0.3; 1.8)	8.5E-01	9.6E-01
Day 4	11	0.1 (-0.2; 0.2)	11	0.1 (0; 0.2)	2.7E-01	9.4E-01
Day 7	11	0.1 (-0.2; 0.1)	11	0.1 (0; 0.2)	7.0E-01	9.6E-01
Day 10	11	0.1 (-0.1; 0.1)	11	0 (0; 0.1)	3.0E-01	9.4E-01
Day 30	10	0 (0; 0.1)	10	0 (0; 0.1)	5.3E-01	9.4E-01
Day 90	11	0 (-0.1; 0.1)	10	0 (0; 0.1)	6.0E-01	9.4E-01
**β-lactamase activity (log10 nmol/min.g)**						
AUC D0D10	11	3.6 (-1.4; 8.1)	11	2.1 (-2.2; 8.7)	2.4E-01	9.4E-01
AUC D0D30	10	12.5 (-6.5; 26.7)	11	6.7 (-1.2; 21.8)	5.6E-01	9.4E-01
Day 4	11	0.4 (-0.3; 0.8)	11	0 (-0.6; 1.4)	2.4E-01	9.4E-01
Day 7	11	0.4 (0; 1.2)	11	0.3 (-0.9; 0.8)	2.9E-01	9.4E-01
Day 10	11	0.5 (-0.2; 1.5)	11	0.3 (-0.1; 1.1)	7.5E-01	9.6E-01
Day 30	10	0.4 (-0.4; 0.8)	11	0.2 (-0.1; 0.6)	3.4E-01	9.4E-01
Day 90	11	0.1 (-0.4; 1)	11	0.2 (-0.2; 0.9)	9.2E-01	>9.9E-01
**Phage microbiome richness (contigs/g)**						
AUC D0D10	10	-1740 (-4190; 1450)	10	-631 (-2470; 2230)	1.1E-01	9.4E-01
AUC D0D30	6	-2790 (-5670; 4740)	8	-630 (-4130; 8050)	2.8E-01	9.4E-01
Day 4	10	-292 (-708; 144)	9	-206 (-388; 219)	2.1E-01	9.4E-01
Day 7	-	-	-	-	-	-
Day 10	10	-200 (-421; 243)	10	-27 (-304; 371)	**4.3E-02**	9.4E-01
Day 30	6	56 (-59; 198)	8	-34 (-233; 206)	2.8E-01	9.4E-01
Day 90	-	-	-	-	-	-
**Phage microbiome structure**						
AUC D0D10	10	13.3 (10.6; 17.3)	10	12.2 (9.7; 19.8)	9.7E-01	>9.9E-01
AUC D0D30	6	32.4 (27.4; 38.4)	8	32 (29.8; 47)	7.5E-01	9.9E-01
Day 4	10	1.3 (0.9; 2.3)	9	1.5 (1; 2.6)	4.5E-01	9.9E-01
Day 7	-	-	-	-	-	-
Day 10	10	1.3 (1; 1.9)	10	1.2 (0.9; 1.6)	2.5E-01	9.9E-01
Day 30	6	1 (0.7; 1.2)	8	1.1 (0.8; 1.3)	2.3E-01	9.9E-01
Day 90	-	-	-	-	-	-
**Fungal load (log10)**						
AUC D0D10	11	10.6 (-9.1; 47.5)	9	2.2 (-6.2; 36.1)	5.5E-01	9.4E-01
AUC D0D30	10	42.4 (-18.9; 114)	9	27.7 (-39.2; 95.8)	4.5E-01	9.4E-01
Day 4	6	1.6 (-0.5; 5.4)	8	0.6 (-0.7; 3.9)	5.7E-01	9.4E-01
Day 7	10	1.4 (-0.4; 5.7)	9	0.9 (-0.6; 3.7)	7.8E-01	9.6E-01
Day 10	11	1.6 (-0.7; 3.6)	9	0.4 (-2.3; 4)	3.7E-01	9.4E-01
Day 30	10	1.2 (-1.8; 4.4)	9	1.2 (-1.5; 4.1)	9.0E-01	1.0E+00
Day 90	11	0.2 (-3.6; 6.8)	9	0.9 (-1; 4)	4.6E-01	9.4E-01

**Fungal microbiome richness (fungal OTUs/g)**						
AUC D0D10	11	-0.8 (-250; 126)	10	49.9 (-227; 181)	5.1E-01	9.4E-01
AUC D0D30	10	-29.9 (-800; 285)	10	153 (-478; 520)	2.8E-01	9.4E-01
Day 4	11	3.5 (-27; 24)	11	2 (-25; 16)	6.4E-01	9.4E-01
Day 7	11	5.5 (-40; 31)	11	6 (-29; 33)	8.3E-01	9.6E-01
Day 10	11	-1 (-12; 18)	11	6.5 (-20; 21)	3.4E-01	9.4E-01
Day 30	10	-3.5 (-23; 15)	11	1.5 (-32; 32)	5.2E-01	9.4E-01
Day 90	11	1.5 (-13; 17)	11	-2 (-36; 28)	7.6E-01	9.6E-01
**Fungal microbiome structure**						
AUC D0D10	11	9.9 (9; 12)	10	10.1 (9; 11.3)	6.0E-01	9.9E-01
AUC D0D30	10	29.2 (26.2; 32.7)	10	30.2 (24.6; 35.1)	2.8E-01	9.9E-01
Day 4	6	1 (0.8; 1.1)	9	1.1 (0.9; 1.1)	9.5E-01	>9.9E-01
Day 7	10	1 (0.9; 1.2)	11	1 (0.8; 1.3)	6.0E-01	9.9E-01
Day 10	11	1 (0.8; 1.1)	10	1 (0.8; 1.3)	7.6E-01	9.9E-01
Day 30	10	1 (0.9; 1.1)	10	1.1 (0.7; 1.2)	4.4E-01	9.9E-01
Day 90	10	1 (0.8; 1.2)	10	1 (0.7; 1.3)	8.5E-01	9.9E-01
**C. albicans DNA concentration (log10)**						
AUC D0D10	11	0.9 (-16.1; 15.3)	9	-1.7 (-22; 10.7)	6.6E-01	9.4E-01
AUC D0D30	10	8.8 (-28.6; 43.6)	9	-14.1 (-58.7; 59.1)	2.1E-01	9.4E-01
Day 4	6	0.7 (-1.4; 1.5)	8	0 (-1.6; 2.4)	6.6E-01	9.4E-01
Day 7	10	-0.2 (-1.3; 1.8)	9	0.1 (-2.7; 1.7)	>9.9E-01	>9.9E-01
Day 10	11	0.1 (-2.8; 1.7)	9	-0.8 (-2.6; 1)	4.1E-01	9.4E-01
Day 30	10	0.4 (-1.1; 1.5)	9	-0.2 (-2; 2.5)	4.0E-01	9.4E-01
Day 90	11	-0.1 (-2.8; 0.8)	9	-0.2 (-1.6; 0.5)	>9.9E-01	>9.9E-01
**Metabolome richness (chemical species/g)**						
AUC D0D10	11	-290 (-4400; 717)	10	-109 (-849; 5720)	6.0E-01	9.4E-01
AUC D0D30	10	-662 (-23400; 1500)	9	-409 (-2860; 18400)	6.6E-01	9.4E-01
Day 4	11	-65 (-394; 117)	11	-21 (-108; 693)	1.3E-01	9.4E-01
Day 7	11	4 (-380; 150)	11	-25 (-67; 515)	8.2E-01	9.6E-01
Day 10	11	-15 (-1260; 117)	11	42.5 (-136; 566)	4.8E-01	9.4E-01
Day 30	10	-41 (-229; 63)	11	-27 (-143; 631)	7.2E-01	9.6E-01
Day 90	11	-28 (-86; 79)	11	-15.5 (-97; 520)	8.1E-01	9.6E-01
**Metabolome structure**						
AUC D0D10	11	11.4 (9; 17.9)	10	11.6 (10.3; 13.9)	8.1E-01	9.9E-01
AUC D0D30	10	33.9 (27.3; 45.2)	9	33.8 (28.3; 41.5)	9.0E-01	1.0E+00
Day 4	11	1.3 (1; 2)	11	1.4 (0.9; 2.1)	5.2E-01	9.9E-01
Day 7	11	1.2 (1; 1.7)	9	1 (0.9; 1.4)	5.0E-02	9.9E-01
Day 10	11	1.1 (0.7; 2.1)	10	1 (0.9; 1.2)	9.0E-02	9.9E-01
Day 30	10	1.1 (0.8; 1.3)	9	1.1 (0.9; 1.6)	6.0E-01	9.9E-01
Day 90	11	1.2 (0.8; 1.3)	10	1.2 (0.8; 1.8)	8.6E-01	9.9E-01
**Cholesterol conversion rate (log10)**						
AUC D0D10	11	-2.2 (-23.8; 8.7)	10	-9.6 (-24.4; -3.2)	**1.3E-02**	9.4E-01
AUC D0D30	10	-5.1 (-35.7; 17.9)	9	-13.6 (-56.2; -2.6)	2.1E-01	9.4E-01
Day 4	11	-0.1 (-3.1; 3.5)	11	-1.8 (-3.3; 0)	**4.7E-02**	9.4E-01
Day 7	11	-0.2 (-3.6; 0.4)	9	-1.2 (-3.6; 1.1)	7.7E-01	9.6E-01
Day 10	11	-0.3 (-3.9; 2.6)	10	-0.1 (-4.3; 0.1)	7.6E-01	9.6E-01

Day 30	10	0 (-1; 0.2)	9	0 (-0.3; 0.1)	4.5E-01	9.4E-01
Day 90	11	0 (-0.2; 0.8)	10	-0.1 (-1.8; 1.8)	2.2E-01	9.4E-01
**Bile acid transformation capacity (log10)**						
AUC D0D10	11	-0.8 (-10.5; 0.7)	10	-1.4 (-5.9; 0.3)	2.5E-01	9.4E-01
AUC D0D30	10	-1 (-12.7; 3.1)	9	-1.1 (-7.1; 1.5)	>9.9E-01	>9.9E-01
Day 4	10	-0.1 (-1.4; 0)	11	-0.2 (-0.7; 0)	1.5E-01	9.4E-01
Day 7	11	-0.1 (-1.2; 0.1)	10	-0.1 (-1; 0.1)	7.6E-01	9.6E-01
Day 10	11	-0.1 (-1.1; 0.1)	10	0 (-0.2; 0.1)	3.9E-01	9.4E-01
Day 30	10	0 (-0.1; 0.4)	9	0 (0; 0.1)	1.6E-01	9.4E-01
Day 90	10	0 (-0.4; 0.4)	10	0 (-0.2; 0.2)	3.9E-01	9.4E-01

Supplementary Table S3. Spearman's correlations of the maximal perturbations of studied gut microbiota and stool components in the 22 healthy volunteers included in the CEREMI trial.

P-values corresponds to the uncorrected test of the Spearman's correlation coefficient to 0. Q-value corresponds to the test of the Spearman's correlation coefficient to 0 after Benjamini & Hochberg's correction. P-values and Q-values in bold represent values below 0.05. Conc., concentration; Cholesterol conv. rate, Cholesterol conversion rate; BA transf. Capacity, Bile acids transformation capacity.

Variable 1	Variable 2	Correlation	P-value	Q-value
Bacterial counts	Bacterial microbiome richness	0.56	**6.7E-03**	2.3E-01
Bacterial counts	Bacterial microbiome structure	0.42	5.6E-02	3.8E-01
Bacterial counts	Enterobacterales counts	0.01	9.8E-01	>9.9E-01
Bacterial counts	Global resistome richness	0.48	**2.6E-02**	3.6E-01
Bacterial counts	B-lactamasome abundance	0.26	2.4E-01	7.3E-01
Bacterial counts	B-lactamase activity	0.26	2.4E-01	7.3E-01
Bacterial counts	Phage microbiome richness	-0.52	**1.7E-02**	3.3E-01
Bacterial counts	Phage microbiome structure	-0.15	5.3E-01	9.4E-01
Bacterial counts	Fungal load	-0.49	**2.6E-02**	3.6E-01
Bacterial counts	Fungal microbiome richness	-0.13	5.6E-01	9.4E-01
Bacterial counts	Fungal microbiome structure	0.01	9.5E-01	>9.9E-01
Bacterial counts	C. albicans DNA conc.	-0.20	3.9E-01	8.5E-01
Bacterial counts	Metabolome richness	-0.05	8.3E-01	9.9E-01
Bacterial counts	Metabolome structure	0.02	9.4E-01	>9.9E-01
Bacterial counts	Cholesterol conv. rate	-0.32	1.5E-01	7.0E-01
Bacterial counts	BA transf. capacity	0.11	6.1E-01	9.4E-01
Bacterial microbiome richness	Bacterial microbiome structure	0.10	6.6E-01	9.7E-01
Bacterial microbiome richness	Enterobacterales counts	-0.29	2.0E-01	7.0E-01
Bacterial microbiome richness	Global resistome richness	0.66	**7.6E-04**	5.2E-02
Bacterial microbiome richness	B-lactamasome abundance	0.28	2.0E-01	7.0E-01
Bacterial microbiome richness	B-lactamase activity	-0.01	9.7E-01	>9.9E-01
Bacterial microbiome richness	Phage microbiome richness	-0.12	5.9E-01	9.4E-01
Bacterial microbiome richness	Phage microbiome structure	0.27	2.4E-01	7.3E-01
Bacterial microbiome richness	Fungal load	-0.44	**4.8E-02**	3.8E-01
Bacterial microbiome richness	Fungal microbiome richness	0.03	8.9E-01	>9.9E-01
Bacterial microbiome richness	Fungal microbiome structure	0.22	3.2E-01	8.1E-01
Bacterial microbiome richness	C. albicans DNA conc.	-0.32	1.5E-01	7.0E-01
Bacterial microbiome richness	Metabolome richness	-0.01	9.5E-01	>9.9E-01
Bacterial microbiome richness	Metabolome structure	0.00	9.9E-01	>9.9E-01
Bacterial microbiome richness	Cholesterol conv. rate	-0.29	1.9E-01	7.0E-01
Bacterial microbiome richness	BA transf. capacity	0.42	5.2E-02	3.8E-01
Bacterial microbiome structure	Enterobacterales counts	-0.11	6.3E-01	9.5E-01
Bacterial microbiome structure	Global resistome richness	0.08	7.1E-01	9.8E-01
Bacterial microbiome structure	B-lactamasome abundance	0.03	8.9E-01	>9.9E-01
Bacterial microbiome structure	B-lactamase activity	0.24	2.7E-01	7.7E-01

**Variable 1**	**Variable 2**	**Correlation**	**P-value**	**Q-value**
Bacterial microbiome structure	Phage microbiome richness	-0.44	**4.8E-02**	3.8E-01
Bacterial microbiome structure	Phage microbiome structure	0.30	1.9E-01	7.0E-01
Bacterial microbiome structure	Fungal load	-0.04	8.8E-01	>9.9E-01
Bacterial microbiome structure	Fungal microbiome richness	-0.13	5.6E-01	9.4E-01
Bacterial microbiome structure	Fungal microbiome structure	0.36	1.0E-01	5.8E-01
Bacterial microbiome structure	C. albicans DNA conc.	-0.09	6.9E-01	9.7E-01
Bacterial microbiome structure	Metabolome richness	0.00	9.9E-01	>9.9E-01
Bacterial microbiome structure	Metabolome structure	0.50	**1.9E-02**	3.3E-01
Bacterial microbiome structure	Cholesterol conv. rate	0.37	9.2E-02	5.6E-01
Bacterial microbiome structure	BA transf. capacity	0.55	**8.6E-03**	2.3E-01
Enterobacterales counts	Phage microbiome richness	-0.06	8.1E-01	9.9E-01
Enterobacterales counts	Phage microbiome structure	-0.33	1.5E-01	7.0E-01
Enterobacterales counts	Global resistome richness	-0.20	3.7E-01	8.4E-01
Enterobacterales counts	B-lactamase activity	0.02	9.5E-01	>9.9E-01
Enterobacterales counts	B-lactamasome abundance	0.08	7.2E-01	9.8E-01
Enterobacterales counts	Fungal load	0.38	9.4E-02	5.6E-01
Enterobacterales counts	Fungal microbiome richness	0.17	4.4E-01	8.8E-01
Enterobacterales counts	Fungal microbiome structure	-0.14	5.4E-01	9.4E-01
Enterobacterales counts	C. albicans DNA conc.	-0.01	9.7E-01	>9.9E-01
Enterobacterales counts	Metabolome richness	0.30	1.7E-01	7.0E-01
Enterobacterales counts	Metabolome structure	-0.11	6.1E-01	9.4E-01
Enterobacterales counts	Cholesterol conv. rate	-0.24	2.8E-01	7.7E-01
Enterobacterales counts	BA transf. capacity	-0.27	2.2E-01	7.3E-01
Global resistome richness	B-lactamase activity	-0.05	8.4E-01	9.9E-01
Global resistome richness	B-lactamasome abundance	-0.15	4.9E-01	9.4E-01
Global resistome richness	Phage microbiome richness	0.07	7.5E-01	9.8E-01
Global resistome richness	Phage microbiome structure	-0.01	9.7E-01	>9.9E-01
Global resistome richness	Fungal load	-0.45	**4.2E-02**	3.8E-01
Global resistome richness	Fungal microbiome richness	-0.06	7.8E-01	9.8E-01
Global resistome richness	Fungal microbiome structure	-0.05	8.2E-01	9.9E-01
Global resistome richness	C. albicans DNA conc.	-0.21	3.6E-01	8.4E-01
Global resistome richness	Metabolome richness	0.23	2.9E-01	7.9E-01
Global resistome richness	Metabolome structure	-0.22	3.1E-01	8.1E-01
Global resistome richness	Cholesterol conv. rate	-0.44	**4.4E-02**	3.8E-01
Global resistome richness	BA transf. capacity	0.06	8.1E-01	9.9E-01
B-lactamasome abundance	B-lactamase activity	-0.15	5.0E-01	9.4E-01
B-lactamasome abundance	Phage microbiome richness	-0.44	5.0E-02	3.8E-01
B-lactamasome abundance	Phage microbiome structure	-0.12	6.1E-01	9.4E-01
B-lactamasome abundance	Fungal load	0.08	7.2E-01	9.8E-01
B-lactamasome abundance	Fungal microbiome richness	0.05	8.3E-01	9.9E-01
B-lactamasome abundance	Fungal microbiome structure	0.22	3.3E-01	8.1E-01
B-lactamasome abundance	C. albicans DNA conc.	0.15	5.0E-01	9.4E-01
B-lactamasome abundance	Metabolome richness	-0.19	4.1E-01	8.8E-01
B-lactamasome abundance	Metabolome structure	0.18	4.3E-01	8.8E-01

**Variable 1**	**Variable 2**	**Correlation**	**P-value**	**Q-value**
B-lactamasome abundance	Cholesterol conv. rate	0.27	2.2E-01	7.3E-01
B-lactamasome abundance	BA transf. capacity	0.11	6.2E-01	9.4E-01
B-lactamase activity	Phage microbiome richness	0.01	9.6E-01	>9.9E-01
B-lactamase activity	Phage microbiome structure	0.09	6.9E-01	9.7E-01
B-lactamase activity	Fungal load	-0.32	1.6E-01	7.0E-01
B-lactamase activity	Fungal microbiome richness	0.33	1.4E-01	7.0E-01
B-lactamase activity	Fungal microbiome structure	0.22	3.3E-01	8.1E-01
B-lactamase activity	C. albicans DNA conc.	0.00	>9.9E-01	>9.9E-01
B-lactamase activity	Metabolome richness	-0.26	2.4E-01	7.3E-01
B-lactamase activity	Metabolome structure	-0.17	4.4E-01	8.8E-01
B-lactamase activity	Cholesterol conv. rate	-0.08	7.2E-01	9.8E-01
B-lactamase activity	BA transf. capacity	-0.05	8.1E-01	9.9E-01
Phage microbiome richness	Phage microbiome structure	-0.01	9.7E-01	>9.9E-01
Phage microbiome richness	Fungal load	0.07	7.8E-01	9.8E-01
Phage microbiome richness	Fungal microbiome richness	0.14	5.4E-01	9.4E-01
Phage microbiome richness	Fungal microbiome structure	-0.18	4.4E-01	8.8E-01
Phage microbiome richness	C. albicans DNA conc.	-0.22	3.6E-01	8.4E-01
Phage microbiome richness	Metabolome richness	0.15	5.1E-01	9.4E-01
Phage microbiome richness	Metabolome structure	-0.25	2.7E-01	7.7E-01
Phage microbiome richness	Cholesterol conv. rate	-0.07	7.6E-01	9.8E-01
Phage microbiome richness	BA transf. capacity	-0.22	3.4E-01	8.1E-01
Phage microbiome structure	Fungal load	0.13	5.8E-01	9.4E-01
Phage microbiome structure	Fungal microbiome richness	-0.07	7.8E-01	9.8E-01
Phage microbiome structure	Fungal microbiome structure	0.10	6.8E-01	9.7E-01
Phage microbiome structure	C. albicans DNA conc.	-0.23	3.3E-01	8.1E-01
Phage microbiome structure	Metabolome richness	0.12	6.1E-01	9.4E-01
Phage microbiome structure	Metabolome structure	0.20	3.9E-01	8.5E-01
Phage microbiome structure	Cholesterol conv. rate	0.33	1.5E-01	7.0E-01
Phage microbiome structure	BA transf. capacity	0.47	**3.4E-02**	3.8E-01
Fungal load	Fungal microbiome richness	0.07	7.5E-01	9.8E-01
Fungal load	Fungal microbiome structure	0.02	9.3E-01	>9.9E-01
Fungal load	C. albicans DNA conc.	0.30	1.8E-01	7.0E-01
Fungal load	Metabolome richness	0.42	5.6E-02	3.8E-01
Fungal load	Metabolome structure	0.17	4.5E-01	8.9E-01
Fungal load	Cholesterol conv. rate	0.24	3.0E-01	7.9E-01
Fungal load	BA transf. capacity	0.12	6.1E-01	9.4E-01
Fungal microbiome richness	Fungal microbiome structure	0.29	2.0E-01	7.0E-01
Fungal microbiome richness	C. albicans DNA conc.	0.29	2.0E-01	7.0E-01
Fungal microbiome richness	Metabolome richness	0.00	9.9E-01	>9.9E-01
Fungal microbiome richness	Metabolome structure	0.18	4.2E-01	8.8E-01
Fungal microbiome richness	Cholesterol conv. rate	0.13	5.8E-01	9.4E-01
Fungal microbiome richness	BA transf. capacity	0.24	2.8E-01	7.7E-01
Fungal microbiome structure	C. albicans DNA conc.	0.15	5.2E-01	9.4E-01
Fungal microbiome structure	Metabolome richness	0.01	9.8E-01	>9.9E-01

**Variable 1**	**Variable 2**	**Correlation**	**P-value**	**Q-value**
Fungal microbiome structure	Metabolome structure	0.51	**1.5E-02**	3.3E-01
Fungal microbiome structure	Cholesterol conv. rate	0.29	1.9E-01	7.0E-01
Fungal microbiome structure	BA transf. capacity	0.44	**4.0E-02**	3.8E-01
C. albicans DNA conc.	Metabolome richness	-0.10	6.7E-01	9.7E-01
C. albicans DNA conc.	Metabolome structure	-0.07	7.6E-01	9.8E-01
C. albicans DNA conc.	Cholesterol conv. rate	0.02	9.5E-01	>9.9E-01
C. albicans DNA conc.	BA transf. capacity	-0.17	4.7E-01	9.1E-01
Metabolome richness	Metabolome structure	-0.01	9.8E-01	>9.9E-01
Metabolome richness	Cholesterol conv. rate	-0.10	6.6E-01	9.7E-01
Metabolome richness	BA transf. capacity	0.10	6.7E-01	9.7E-01
Metabolome structure	Cholesterol conv. rate	0.71	**3.5E-04**	**4.7E-02**
Metabolome structure	BA transf. capacity	0.58	**5.5E-03**	2.3E-01
Cholesterol conv. rate	BA transf. capacity	0.37	9.3E-02	5.6E-01

Supplementary Table S4. Spearman's correlations of the maximal resiliences of studied gut microbiota and stool components in the 22 healthy volunteers included in the CEREMI trial.

P-values corresponds to the uncorrected test of the Spearman's correlation coefficient to 0. Q-value corresponds to the test of the Spearman's correlation coefficient to 0 after Benjamini & Hochberg's correction. P-values in bold represent values below 0.05. Conc., concentration; Cholesterol conv. rate, Cholesterol conversion rate; BA transf. Capacity, Bile acids transformation capacity.

Variable 1	Variable 2	Correlation	P-value	Q-value
Bacterial counts	Bacterial microbiome richness	0.46	**3.7E-02**	7.3E-01
Bacterial counts	Bacterial microbiome structure	0.48	**3.0E-02**	7.3E-01
Bacterial counts	Enterobacterales counts	-0.24	3.0E-01	9.0E-01
Bacterial counts	Global resistome richness	0.35	1.3E-01	7.8E-01
Bacterial counts	B-lactamasome abundance	-0.16	4.8E-01	9.0E-01
Bacterial counts	B-lactamase activity	0.20	4.0E-01	9.0E-01
Bacterial counts	Phage microbiome richness	-0.14	6.4E-01	9.0E-01
Bacterial counts	Phage microbiome structure	0.41	7.1E-02	7.4E-01
Bacterial counts	Fungal load	0.11	6.5E-01	9.0E-01
Bacterial counts	Fungal microbiome richness	0.23	3.3E-01	9.0E-01
Bacterial counts	Fungal microbiome structure	0.09	7.0E-01	9.0E-01
Bacterial counts	C. albicans DNA conc.	0.07	7.8E-01	9.0E-01
Bacterial counts	Metabolome richness	-0.20	4.0E-01	9.0E-01
Bacterial counts	Metabolome structure	-0.09	7.1E-01	9.0E-01
Bacterial counts	Cholesterol conv. rate	0.06	8.1E-01	9.0E-01
Bacterial counts	BA transf. capacity	-0.07	7.7E-01	9.0E-01
Bacterial microbiome richness	Bacterial microbiome structure	0.08	7.2E-01	9.0E-01
Bacterial microbiome richness	Enterobacterales counts	-0.35	1.2E-01	7.8E-01
Bacterial microbiome richness	Global resistome richness	0.31	1.7E-01	8.7E-01
Bacterial microbiome richness	B-lactamasome abundance	-0.10	6.6E-01	9.0E-01
Bacterial microbiome richness	B-lactamase activity	0.45	**3.7E-02**	7.3E-01
Bacterial microbiome richness	Phage microbiome richness	-0.22	4.4E-01	9.0E-01
Bacterial microbiome richness	Phage microbiome structure	-0.14	5.5E-01	9.0E-01
Bacterial microbiome richness	Fungal load	0.17	4.5E-01	9.0E-01
Bacterial microbiome richness	Fungal microbiome richness	0.18	4.2E-01	9.0E-01
Bacterial microbiome richness	Fungal microbiome structure	0.42	5.7E-02	7.4E-01
Bacterial microbiome richness	C. albicans DNA conc.	-0.07	7.5E-01	9.0E-01
Bacterial microbiome richness	Metabolome richness	-0.38	8.9E-02	7.8E-01
Bacterial microbiome richness	Metabolome structure	-0.06	8.1E-01	9.0E-01
Bacterial microbiome richness	Cholesterol conv. rate	0.19	4.0E-01	9.0E-01
Bacterial microbiome richness	BA transf. capacity	-0.31	1.8E-01	8.9E-01
Bacterial microbiome structure	Enterobacterales counts	-0.17	4.4E-01	9.0E-01
Bacterial microbiome structure	Global resistome richness	0.16	4.6E-01	9.0E-01
Bacterial microbiome structure	B-lactamasome abundance	0.04	8.8E-01	9.3E-01
Bacterial microbiome structure	B-lactamase activity	-0.13	5.7E-01	9.0E-01
Bacterial microbiome structure	Phage microbiome richness	-0.08	7.8E-01	9.0E-01

**Variable 1**	**Variable 2**	**Correlation**	**P-value**	**Q-value**
Bacterial microbiome structure	Phage microbiome structure	0.53	**1.4E-02**	6.9E-01
Bacterial microbiome structure	Fungal load	0.07	7.6E-01	9.0E-01
Bacterial microbiome structure	Fungal microbiome richness	-0.15	5.1E-01	9.0E-01
Bacterial microbiome structure	Fungal microbiome structure	0.05	8.5E-01	9.2E-01
Bacterial microbiome structure	C. albicans DNA conc.	-0.12	6.1E-01	9.0E-01
Bacterial microbiome structure	Metabolome richness	-0.10	6.7E-01	9.0E-01
Bacterial microbiome structure	Metabolome structure	0.09	7.0E-01	9.0E-01
Bacterial microbiome structure	Cholesterol conv. rate	-0.06	7.9E-01	9.0E-01
Bacterial microbiome structure	BA transf. capacity	-0.16	4.8E-01	9.0E-01
Enterobacterales counts	Phage microbiome richness	-0.24	4.2E-01	9.0E-01
Enterobacterales counts	Phage microbiome structure	0.19	4.1E-01	9.0E-01
Enterobacterales counts	Global resistome richness	-0.40	6.6E-02	7.4E-01
Enterobacterales counts	B-lactamase activity	-0.10	6.5E-01	9.0E-01
Enterobacterales counts	B-lactamasome abundance	0.52	**1.5E-02**	6.9E-01
Enterobacterales counts	Fungal load	-0.20	3.8E-01	9.0E-01
Enterobacterales counts	Fungal microbiome richness	-0.11	6.3E-01	9.0E-01
Enterobacterales counts	Fungal microbiome structure	-0.32	1.6E-01	8.7E-01
Enterobacterales counts	C. albicans DNA conc.	-0.02	9.4E-01	9.4E-01
Enterobacterales counts	Metabolome richness	0.17	4.7E-01	9.0E-01
Enterobacterales counts	Metabolome structure	-0.55	**1.1E-02**	6.9E-01
Enterobacterales counts	Cholesterol conv. rate	-0.17	4.6E-01	9.0E-01
Enterobacterales counts	BA transf. capacity	-0.11	6.4E-01	9.0E-01
Global resistome richness	B-lactamase activity	-0.18	4.3E-01	9.0E-01
Global resistome richness	B-lactamasome abundance	-0.22	3.3E-01	9.0E-01
Global resistome richness	Phage microbiome richness	-0.32	2.7E-01	9.0E-01
Global resistome richness	Phage microbiome structure	0.09	6.9E-01	9.0E-01
Global resistome richness	Fungal load	-0.04	8.5E-01	9.2E-01
Global resistome richness	Fungal microbiome richness	0.21	3.6E-01	9.0E-01
Global resistome richness	Fungal microbiome structure	0.19	4.0E-01	9.0E-01
Global resistome richness	C. albicans DNA conc.	-0.11	6.4E-01	9.0E-01
Global resistome richness	Metabolome richness	-0.23	3.1E-01	9.0E-01
Global resistome richness	Metabolome structure	0.12	5.9E-01	9.0E-01
Global resistome richness	Cholesterol conv. rate	0.25	2.7E-01	9.0E-01
Global resistome richness	BA transf. capacity	0.16	4.8E-01	9.0E-01
B-lactamasome abundance	B-lactamase activity	-0.11	6.1E-01	9.0E-01
B-lactamasome abundance	Phage microbiome richness	0.05	8.8E-01	9.3E-01
B-lactamasome abundance	Phage microbiome structure	0.05	8.2E-01	9.1E-01
B-lactamasome abundance	Fungal load	-0.19	4.1E-01	9.0E-01
B-lactamasome abundance	Fungal microbiome richness	0.18	4.4E-01	9.0E-01
B-lactamasome abundance	Fungal microbiome structure	-0.11	6.4E-01	9.0E-01
B-lactamasome abundance	C. albicans DNA conc.	0.26	2.6E-01	9.0E-01
B-lactamasome abundance	Metabolome richness	0.22	3.4E-01	9.0E-01
B-lactamasome abundance	Metabolome structure	-0.23	3.2E-01	9.0E-01
B-lactamasome abundance	Cholesterol conv. rate	-0.47	**3.3E-02**	7.3E-01

**Variable 1**	**Variable 2**	**Correlation**	**P-value**	**Q-value**
B-lactamasome abundance	BA transf. capacity	0.26	2.6E-01	9.0E-01
B-lactamase activity	Phage microbiome richness	0.17	5.5E-01	9.0E-01
B-lactamase activity	Phage microbiome structure	-0.22	3.4E-01	9.0E-01
B-lactamase activity	Fungal load	0.20	3.8E-01	9.0E-01
B-lactamase activity	Fungal microbiome richness	-0.03	9.0E-01	9.4E-01
B-lactamase activity	Fungal microbiome structure	0.07	7.8E-01	9.0E-01
B-lactamase activity	C. albicans DNA conc.	-0.37	9.9E-02	7.8E-01
B-lactamase activity	Metabolome richness	-0.18	4.4E-01	9.0E-01
B-lactamase activity	Metabolome structure	0.08	7.4E-01	9.0E-01
B-lactamase activity	Cholesterol conv. rate	-0.27	2.4E-01	9.0E-01
B-lactamase activity	BA transf. capacity	-0.35	1.2E-01	7.8E-01
Phage microbiome richness	Phage microbiome structure	-0.39	1.7E-01	8.7E-01
Phage microbiome richness	Fungal load	0.17	5.6E-01	9.0E-01
Phage microbiome richness	Fungal microbiome richness	-0.09	7.7E-01	9.0E-01
Phage microbiome richness	Fungal microbiome structure	-0.03	9.3E-01	9.4E-01
Phage microbiome richness	C. albicans DNA conc.	0.31	2.7E-01	9.0E-01
Phage microbiome richness	Metabolome richness	0.45	1.1E-01	7.8E-01
Phage microbiome richness	Metabolome structure	-0.17	5.5E-01	9.0E-01
Phage microbiome richness	Cholesterol conv. rate	-0.52	6.2E-02	7.4E-01
Phage microbiome richness	BA transf. capacity	0.23	4.4E-01	9.0E-01
Phage microbiome structure	Fungal load	0.13	5.7E-01	9.0E-01
Phage microbiome structure	Fungal microbiome richness	-0.27	2.5E-01	9.0E-01
Phage microbiome structure	Fungal microbiome structure	-0.17	4.8E-01	9.0E-01
Phage microbiome structure	C. albicans DNA conc.	0.04	8.7E-01	9.3E-01
Phage microbiome structure	Metabolome richness	0.07	7.7E-01	9.0E-01
Phage microbiome structure	Metabolome structure	0.02	9.4E-01	9.4E-01
Phage microbiome structure	Cholesterol conv. rate	-0.02	9.3E-01	9.4E-01
Phage microbiome structure	BA transf. capacity	-0.15	5.4E-01	9.0E-01
Fungal load	Fungal microbiome richness	0.09	6.9E-01	9.0E-01
Fungal load	Fungal microbiome structure	0.16	4.8E-01	9.0E-01
Fungal load	C. albicans DNA conc.	-0.40	7.0E-02	7.4E-01
Fungal load	Metabolome richness	0.09	7.1E-01	9.0E-01
Fungal load	Metabolome structure	0.08	7.2E-01	9.0E-01
Fungal load	Cholesterol conv. rate	-0.23	3.2E-01	9.0E-01
Fungal load	BA transf. capacity	0.28	2.2E-01	9.0E-01
Fungal microbiome richness	Fungal microbiome structure	0.20	3.9E-01	9.0E-01
Fungal microbiome richness	C. albicans DNA conc.	0.19	4.1E-01	9.0E-01
Fungal microbiome richness	Metabolome richness	-0.16	5.0E-01	9.0E-01
Fungal microbiome richness	Metabolome structure	-0.32	1.5E-01	8.7E-01
Fungal microbiome richness	Cholesterol conv. rate	-0.38	9.2E-02	7.8E-01
Fungal microbiome richness	BA transf. capacity	0.39	8.4E-02	7.8E-01
Fungal microbiome structure	C. albicans DNA conc.	0.04	8.5E-01	9.2E-01
Fungal microbiome structure	Metabolome richness	0.06	8.1E-01	9.0E-01
Fungal microbiome structure	Metabolome structure	0.10	6.7E-01	9.0E-01

Variable 1	Variable 2	Correlation	P-value	Q-value
Fungal microbiome structure	Cholesterol conv. rate	0.13	5.8E-01	9.0E-01
Fungal microbiome structure	BA transf. capacity	-0.10	6.8E-01	9.0E-01
C. albicans DNA conc.	Metabolome richness	0.41	6.2E-02	7.4E-01
C. albicans DNA conc.	Metabolome structure	-0.02	9.4E-01	9.4E-01
C. albicans DNA conc.	Cholesterol conv. rate	0.10	6.5E-01	9.0E-01
C. albicans DNA conc.	BA transf. capacity	0.22	3.4E-01	9.0E-01
Metabolome richness	Metabolome structure	0.11	6.4E-01	9.0E-01
Metabolome richness	Cholesterol conv. rate	-0.03	9.1E-01	9.4E-01
Metabolome richness	BA transf. capacity	0.14	5.4E-01	9.0E-01
Metabolome structure	Cholesterol conv. rate	0.14	5.4E-01	9.0E-01
Metabolome structure	BA transf. capacity	0.11	6.5E-01	9.0E-01
Cholesterol conv. rate	BA transf. capacity	-0.36	1.1E-01	7.8E-01

Supplementary Table S5. Spearman's correlations between the baseline characteristics and the maximal perturbations of the studied gut microbiota and gut components in the 22 healthy volunteers included in the CEREMI trial.

P-values corresponds to the uncorrected test of the Spearman's correlation coefficient to 0. Q-value corresponds to the test of the Spearman's correlation coefficient to 0 after Benjamini & Hochberg's correction. P-values in bold represent values below 0.05. Conc., concentration; Cholesterol conv. rate, Cholesterol conversion rate; BA transf. Capacity, Bile acids transformation capacity.

Baseline variable	Perturbation variable	Correlation	P-value	Q-value
Bacterial counts	Bacterial counts	0.18	4.2E-01	9.9E-01
Bacterial counts	Bacterial microbiome richness	-0.02	9.2E-01	>9.9E-01
Bacterial counts	Bacterial microbiome structure	0.01	9.6E-01	>9.9E-01
Bacterial counts	Enterobacterales counts	0.14	5.4E-01	9.9E-01
Bacterial counts	Global resistome richness	-0.22	3.3E-01	9.9E-01
Bacterial counts	B-lactamasome abundance	0.41	6.2E-02	9.9E-01
Bacterial counts	B-lactamase activity	0.21	3.5E-01	9.9E-01
Bacterial counts	Phage microbiome richness	0.04	8.7E-01	9.9E-01
Bacterial counts	Phage microbiome structure	-0.27	2.3E-01	9.9E-01
Bacterial counts	Fungal load	0.05	8.3E-01	9.9E-01
Bacterial counts	Fungal microbiome richness	0.04	8.7E-01	9.9E-01
Bacterial counts	Fungal microbiome structure	-0.19	4.0E-01	9.9E-01
Bacterial counts	C. albicans DNA conc.	-0.23	3.2E-01	9.9E-01
Bacterial counts	Metabolome richness	-0.23	3.0E-01	9.9E-01
Bacterial counts	Metabolome structure	-0.03	8.9E-01	9.9E-01
Bacterial counts	Cholesterol conv. rate	0.07	7.5E-01	9.9E-01
Bacterial counts	BA transf. capacity	0.01	9.7E-01	>9.9E-01
Bacterial microbiome richness	Bacterial counts	-0.23	2.9E-01	9.9E-01
Bacterial microbiome richness	Bacterial microbiome richness	-0.11	6.1E-01	9.9E-01
Bacterial microbiome richness	Bacterial microbiome structure	-0.08	7.3E-01	9.9E-01
Bacterial microbiome richness	Enterobacterales counts	-0.14	5.3E-01	9.9E-01
Bacterial microbiome richness	Global resistome richness	-0.27	2.2E-01	9.9E-01
Bacterial microbiome richness	B-lactamasome abundance	0.22	3.3E-01	9.9E-01
Bacterial microbiome richness	B-lactamase activity	-0.35	1.1E-01	9.9E-01
Bacterial microbiome richness	Phage microbiome richness	0.18	4.4E-01	9.9E-01
Bacterial microbiome richness	Phage microbiome structure	-0.04	8.5E-01	9.9E-01
Bacterial microbiome richness	Fungal load	0.00	9.8E-01	>9.9E-01
Bacterial microbiome richness	Fungal microbiome richness	-0.18	4.2E-01	9.9E-01
Bacterial microbiome richness	Fungal microbiome structure	-0.05	8.2E-01	9.9E-01
Bacterial microbiome richness	C. albicans DNA conc.	-0.37	1.0E-01	9.9E-01
Bacterial microbiome richness	Metabolome richness	-0.10	6.6E-01	9.9E-01
Bacterial microbiome richness	Metabolome structure	0.18	4.1E-01	9.9E-01
Bacterial microbiome richness	Cholesterol conv. rate	0.44	**4.2E-02**	9.9E-01
Bacterial microbiome richness	BA transf. capacity	0.12	6.1E-01	9.9E-01
Enterobacterales counts	Bacterial counts	0.05	8.2E-01	9.9E-01

**Baseline variable**	**Perturbation variable**	**Correlation**	**P-value**	**Q-value**
Enterobacterales counts	Bacterial microbiome richness	0.29	2.0E-01	9.9E-01
Enterobacterales counts	Bacterial microbiome structure	-0.33	1.4E-01	9.9E-01
Enterobacterales counts	Enterobacterales counts	0.18	4.3E-01	9.9E-01
Enterobacterales counts	Global resistome richness	0.20	3.8E-01	9.9E-01
Enterobacterales counts	B-lactamasome abundance	0.05	8.2E-01	9.9E-01
Enterobacterales counts	B-lactamase activity	0.10	6.6E-01	9.9E-01
Enterobacterales counts	Phage microbiome richness	0.11	6.4E-01	9.9E-01
Enterobacterales counts	Phage microbiome structure	-0.08	7.4E-01	9.9E-01
Enterobacterales counts	Fungal load	-0.32	1.6E-01	9.9E-01
Enterobacterales counts	Fungal microbiome richness	0.12	5.9E-01	9.9E-01
Enterobacterales counts	Fungal microbiome structure	-0.18	4.4E-01	9.9E-01
Enterobacterales counts	C. albicans DNA conc.	-0.17	4.6E-01	9.9E-01
Enterobacterales counts	Metabolome richness	-0.21	3.5E-01	9.9E-01
Enterobacterales counts	Metabolome structure	-0.16	4.8E-01	9.9E-01
Enterobacterales counts	Cholesterol conv. rate	-0.24	2.9E-01	9.9E-01
Enterobacterales counts	BA transf. capacity	-0.06	8.0E-01	9.9E-01
Global resistome richness	Bacterial counts	-0.16	4.8E-01	9.9E-01
Global resistome richness	Bacterial microbiome richness	-0.06	7.8E-01	9.9E-01
Global resistome richness	Bacterial microbiome structure	0.07	7.4E-01	9.9E-01
Global resistome richness	Enterobacterales counts	-0.16	4.7E-01	9.9E-01
Global resistome richness	Global resistome richness	-0.19	4.0E-01	9.9E-01
Global resistome richness	B-lactamasome abundance	0.15	5.1E-01	9.9E-01
Global resistome richness	B-lactamase activity	-0.22	3.2E-01	9.9E-01
Global resistome richness	Phage microbiome richness	0.21	3.6E-01	9.9E-01
Global resistome richness	Phage microbiome structure	0.09	7.0E-01	9.9E-01
Global resistome richness	Fungal load	-0.06	8.0E-01	9.9E-01
Global resistome richness	Fungal microbiome richness	-0.16	4.8E-01	9.9E-01
Global resistome richness	Fungal microbiome structure	-0.12	5.9E-01	9.9E-01
Global resistome richness	C. albicans DNA conc.	-0.57	**7.5E-03**	6.5E-01
Global resistome richness	Metabolome richness	-0.17	4.6E-01	9.9E-01
Global resistome richness	Metabolome structure	0.36	1.0E-01	9.9E-01
Global resistome richness	Cholesterol conv. rate	0.54	**8.8E-03**	6.5E-01
Global resistome richness	BA transf. capacity	0.24	2.8E-01	9.9E-01
B-lactamasome abundance	Bacterial counts	-0.07	7.5E-01	9.9E-01
B-lactamasome abundance	Bacterial microbiome richness	0.15	5.1E-01	9.9E-01
B-lactamasome abundance	Bacterial microbiome structure	-0.13	5.5E-01	9.9E-01
B-lactamasome abundance	Enterobacterales counts	0.15	4.9E-01	9.9E-01
B-lactamasome abundance	Global resistome richness	0.35	1.1E-01	9.9E-01
B-lactamasome abundance	B-lactamasome abundance	-0.16	4.8E-01	9.9E-01
B-lactamasome abundance	B-lactamase activity	0.07	7.7E-01	9.9E-01
B-lactamasome abundance	Phage microbiome richness	0.14	5.6E-01	9.9E-01
B-lactamasome abundance	Phage microbiome structure	-0.04	8.5E-01	9.9E-01
B-lactamasome abundance	Fungal load	-0.04	8.7E-01	9.9E-01
B-lactamasome abundance	Fungal microbiome richness	0.34	1.2E-01	9.9E-01

Baseline variable	Perturbation variable	Correlation	P-value	Q-value
B-lactamasome abundance	Fungal microbiome structure	0.04	8.5E-01	9.9E-01
B-lactamasome abundance	C. albicans DNA conc.	0.32	1.5E-01	9.9E-01
B-lactamasome abundance	Metabolome richness	-0.01	9.8E-01	>9.9E-01
B-lactamasome abundance	Metabolome structure	-0.09	6.9E-01	9.9E-01
B-lactamasome abundance	Cholesterol conv. rate	-0.28	2.0E-01	9.9E-01
B-lactamasome abundance	BA transf. capacity	-0.02	9.3E-01	>9.9E-01
B-lactamase activity	Bacterial counts	-0.27	2.2E-01	9.9E-01
B-lactamase activity	Bacterial microbiome richness	-0.25	2.6E-01	9.9E-01
B-lactamase activity	Bacterial microbiome structure	-0.26	2.4E-01	9.9E-01
B-lactamase activity	Enterobacterales counts	-0.26	2.4E-01	9.9E-01
B-lactamase activity	Global resistome richness	-0.41	6.0E-02	9.9E-01
B-lactamase activity	B-lactamasome abundance	0.09	6.9E-01	9.9E-01
B-lactamase activity	B-lactamase activity	-0.14	5.3E-01	9.9E-01
B-lactamase activity	Phage microbiome richness	0.05	8.4E-01	9.9E-01
B-lactamase activity	Phage microbiome structure	0.10	6.6E-01	9.9E-01
B-lactamase activity	Fungal load	0.29	2.1E-01	9.9E-01
B-lactamase activity	Fungal microbiome richness	0.07	7.4E-01	9.9E-01
B-lactamase activity	Fungal microbiome structure	-0.36	9.6E-02	9.9E-01
B-lactamase activity	C. albicans DNA conc.	-0.06	8.1E-01	9.9E-01
B-lactamase activity	Metabolome richness	-0.24	2.7E-01	9.9E-01
B-lactamase activity	Metabolome structure	0.07	7.6E-01	9.9E-01
B-lactamase activity	Cholesterol conv. rate	0.30	1.8E-01	9.9E-01
B-lactamase activity	BA transf. capacity	0.00	9.9E-01	>9.9E-01
Phage microbiome richness	Bacterial counts	-0.31	1.7E-01	9.9E-01
Phage microbiome richness	Bacterial microbiome richness	-0.26	2.6E-01	9.9E-01
Phage microbiome richness	Bacterial microbiome structure	0.03	8.9E-01	9.9E-01
Phage microbiome richness	Enterobacterales counts	-0.34	1.3E-01	9.9E-01
Phage microbiome richness	Global resistome richness	-0.18	4.4E-01	9.9E-01
Phage microbiome richness	B-lactamasome abundance	-0.11	6.2E-01	9.9E-01
Phage microbiome richness	B-lactamase activity	-0.02	9.2E-01	>9.9E-01
Phage microbiome richness	Phage microbiome richness	0.05	8.1E-01	9.9E-01
Phage microbiome richness	Phage microbiome structure	0.20	3.8E-01	9.9E-01
Phage microbiome richness	Fungal load	0.08	7.4E-01	9.9E-01
Phage microbiome richness	Fungal microbiome richness	-0.46	**3.5E-02**	9.9E-01
Phage microbiome richness	Fungal microbiome structure	-0.26	2.6E-01	9.9E-01
Phage microbiome richness	C. albicans DNA conc.	-0.22	3.4E-01	9.9E-01
Phage microbiome richness	Metabolome richness	-0.18	4.5E-01	9.9E-01
Phage microbiome richness	Metabolome structure	-0.21	3.5E-01	9.9E-01
Phage microbiome richness	Cholesterol conv. rate	0.19	4.0E-01	9.9E-01
Phage microbiome richness	BA transf. capacity	-0.10	6.7E-01	9.9E-01
Fungal load	Bacterial counts	0.13	5.9E-01	9.9E-01
Fungal load	Bacterial microbiome richness	-0.03	8.9E-01	9.9E-01
Fungal load	Bacterial microbiome structure	0.09	7.1E-01	9.9E-01
Fungal load	Enterobacterales counts	0.21	3.5E-01	9.9E-01

**Baseline variable**	**Perturbation variable**	**Correlation**	**P-value**	**Q-value**
Fungal load	Global resistome richness	0.06	8.1E-01	9.9E-01
Fungal load	B-lactamasome abundance	0.39	8.4E-02	9.9E-01
Fungal load	B-lactamase activity	-0.08	7.5E-01	9.9E-01
Fungal load	Phage microbiome richness	-0.21	3.8E-01	9.9E-01
Fungal load	Phage microbiome structure	-0.02	9.2E-01	>9.9E-01
Fungal load	Fungal load	0.05	8.5E-01	9.9E-01
Fungal load	Fungal microbiome richness	-0.14	5.5E-01	9.9E-01
Fungal load	Fungal microbiome structure	-0.02	9.3E-01	>9.9E-01
Fungal load	C. albicans DNA conc.	-0.04	8.8E-01	9.9E-01
Fungal load	Metabolome richness	-0.04	8.7E-01	9.9E-01
Fungal load	Metabolome structure	-0.01	9.6E-01	>9.9E-01
Fungal load	Cholesterol conv. rate	0.26	2.5E-01	9.9E-01
Fungal load	BA transf. capacity	-0.18	4.2E-01	9.9E-01
Fungal microbiome richness	Bacterial counts	-0.07	7.6E-01	9.9E-01
Fungal microbiome richness	Bacterial microbiome richness	0.01	9.8E-01	>9.9E-01
Fungal microbiome richness	Bacterial microbiome structure	0.08	7.1E-01	9.9E-01
Fungal microbiome richness	Enterobacterales counts	-0.12	6.0E-01	9.9E-01
Fungal microbiome richness	Global resistome richness	-0.01	9.7E-01	>9.9E-01
Fungal microbiome richness	B-lactamasome abundance	0.17	4.4E-01	9.9E-01
Fungal microbiome richness	B-lactamase activity	0.30	1.8E-01	9.9E-01
Fungal microbiome richness	Phage microbiome richness	-0.11	6.4E-01	9.9E-01
Fungal microbiome richness	Phage microbiome structure	0.00	>9.9E-01	>9.9E-01
Fungal microbiome richness	Fungal load	-0.20	3.8E-01	9.9E-01
Fungal microbiome richness	Fungal microbiome richness	0.21	3.4E-01	9.9E-01
Fungal microbiome richness	Fungal microbiome structure	0.32	1.5E-01	9.9E-01
Fungal microbiome richness	C. albicans DNA conc.	0.00	9.9E-01	>9.9E-01
Fungal microbiome richness	Metabolome richness	-0.05	8.2E-01	9.9E-01
Fungal microbiome richness	Metabolome structure	0.26	2.5E-01	9.9E-01
Fungal microbiome richness	Cholesterol conv. rate	0.22	3.3E-01	9.9E-01
Fungal microbiome richness	BA transf. capacity	0.12	6.1E-01	9.9E-01
C. albicans DNA conc.	Bacterial counts	-0.41	6.4E-02	9.9E-01
C. albicans DNA conc.	Bacterial microbiome richness	-0.09	7.0E-01	9.9E-01
C. albicans DNA conc.	Bacterial microbiome structure	-0.18	4.3E-01	9.9E-01
C. albicans DNA conc.	Enterobacterales counts	-0.04	8.8E-01	9.9E-01
C. albicans DNA conc.	Global resistome richness	-0.23	3.1E-01	9.9E-01
C. albicans DNA conc.	B-lactamasome abundance	0.04	8.7E-01	9.9E-01
C. albicans DNA conc.	B-lactamase activity	-0.11	6.5E-01	9.9E-01
C. albicans DNA conc.	Phage microbiome richness	0.47	**3.8E-02**	9.9E-01
C. albicans DNA conc.	Phage microbiome structure	0.14	5.5E-01	9.9E-01
C. albicans DNA conc.	Fungal load	0.26	2.6E-01	9.9E-01
C. albicans DNA conc.	Fungal microbiome richness	0.11	6.3E-01	9.9E-01
C. albicans DNA conc.	Fungal microbiome structure	0.05	8.2E-01	9.9E-01
C. albicans DNA conc.	C. albicans DNA conc.	0.05	8.1E-01	9.9E-01
C. albicans DNA conc.	Metabolome richness	0.09	7.0E-01	9.9E-01

Baseline variable	Perturbation variable	Correlation	P-value	Q-value
C. albicans DNA conc.	Metabolome structure	-0.15	5.2E-01	9.9E-01
C. albicans DNA conc.	Cholesterol conv. rate	0.16	4.8E-01	9.9E-01
C. albicans DNA conc.	BA transf. capacity	-0.12	6.0E-01	9.9E-01
Metabolome richness	Bacterial counts	-0.03	8.8E-01	9.9E-01
Metabolome richness	Bacterial microbiome richness	-0.27	2.2E-01	9.9E-01
Metabolome richness	Bacterial microbiome structure	0.18	4.1E-01	9.9E-01
Metabolome richness	Enterobacterales counts	-0.12	5.8E-01	9.9E-01
Metabolome richness	Global resistome richness	-0.09	6.9E-01	9.9E-01
Metabolome richness	B-lactamasome abundance	-0.02	9.3E-01	>9.9E-01
Metabolome richness	B-lactamase activity	0.19	4.1E-01	9.9E-01
Metabolome richness	Phage microbiome richness	0.16	4.9E-01	9.9E-01
Metabolome richness	Phage microbiome structure	-0.07	7.5E-01	9.9E-01
Metabolome richness	Fungal load	0.07	7.8E-01	9.9E-01
Metabolome richness	Fungal microbiome richness	0.27	2.2E-01	9.9E-01
Metabolome richness	Fungal microbiome structure	0.10	6.7E-01	9.9E-01
Metabolome richness	C. albicans DNA conc.	0.11	6.4E-01	9.9E-01
Metabolome richness	Metabolome richness	0.21	3.4E-01	9.9E-01
Metabolome richness	Metabolome structure	0.29	1.9E-01	9.9E-01
Metabolome richness	Cholesterol conv. rate	0.35	1.1E-01	9.9E-01
Metabolome richness	BA transf. capacity	0.04	8.6E-01	9.9E-01
Cholesterol conv. rate	Bacterial counts	-0.20	3.6E-01	9.9E-01
Cholesterol conv. rate	Bacterial microbiome richness	-0.17	4.4E-01	9.9E-01
Cholesterol conv. rate	Bacterial microbiome structure	-0.05	8.3E-01	9.9E-01
Cholesterol conv. rate	Enterobacterales counts	-0.03	9.0E-01	9.9E-01
Cholesterol conv. rate	Global resistome richness	-0.41	5.7E-02	9.9E-01
Cholesterol conv. rate	B-lactamasome abundance	0.42	5.0E-02	9.9E-01
Cholesterol conv. rate	B-lactamase activity	0.04	8.4E-01	9.9E-01
Cholesterol conv. rate	Phage microbiome richness	0.23	3.1E-01	9.9E-01
Cholesterol conv. rate	Phage microbiome structure	-0.29	2.0E-01	9.9E-01
Cholesterol conv. rate	Fungal load	0.17	4.7E-01	9.9E-01
Cholesterol conv. rate	Fungal microbiome richness	0.09	6.9E-01	9.9E-01
Cholesterol conv. rate	Fungal microbiome structure	0.18	4.2E-01	9.9E-01
Cholesterol conv. rate	C. albicans DNA conc.	-0.01	9.8E-01	>9.9E-01
Cholesterol conv. rate	Metabolome richness	-0.36	9.7E-02	9.9E-01
Cholesterol conv. rate	Metabolome structure	0.15	5.0E-01	9.9E-01
Cholesterol conv. rate	Cholesterol conv. rate	0.29	1.9E-01	9.9E-01
Cholesterol conv. rate	BA transf. capacity	-0.01	9.7E-01	>9.9E-01
BA transf. capacity	Bacterial counts	-0.08	7.2E-01	9.9E-01
BA transf. capacity	Bacterial microbiome richness	0.23	3.0E-01	9.9E-01
BA transf. capacity	Bacterial microbiome structure	0.13	5.6E-01	9.9E-01
BA transf. capacity	Enterobacterales counts	-0.16	4.8E-01	9.9E-01
BA transf. capacity	Global resistome richness	-0.04	8.8E-01	9.9E-01
BA transf. capacity	B-lactamasome abundance	-0.01	9.6E-01	>9.9E-01
BA transf. capacity	B-lactamase activity	0.12	6.0E-01	9.9E-01

**Baseline variable**	**Perturbation variable**	**Correlation**	**P-value**	**Q-value**
BA transf. capacity	Phage microbiome richness	0.14	5.3E-01	9.9E-01
BA transf. capacity	Phage microbiome structure	0.17	4.7E-01	9.9E-01
BA transf. capacity	Fungal load	-0.04	8.6E-01	9.9E-01
BA transf. capacity	Fungal microbiome richness	0.32	1.5E-01	9.9E-01
BA transf. capacity	Fungal microbiome structure	0.17	4.5E-01	9.9E-01
BA transf. capacity	C. albicans DNA conc.	-0.41	7.0E-02	9.9E-01
BA transf. capacity	Metabolome richness	-0.01	9.8E-01	>9.9E-01
BA transf. capacity	Metabolome structure	0.30	1.8E-01	9.9E-01
BA transf. capacity	Cholesterol conv. rate	0.12	5.8E-01	9.9E-01
BA transf. capacity	BA transf. capacity	0.62	**2.7E-03**	5.9E-01

Supplementary Table S6. Spearman's correlations between the baseline characteristics and the maximal resiliences of the studied gut microbiota and gut components in the 22 healthy volunteers included in the CEREMI trial.

P-values corresponds to the uncorrected test of the Spearman's correlation coefficient to 0. Q-value corresponds to the test of the Spearman's correlation coefficient to 0 after Benjamini & Hochberg's correction. P-values in bold represent values below 0.05. Conc., concentration; Cholesterol conv. rate, Cholesterol conversion rate; BA transf. Capacity, Bile acids transformation capacity.

Baseline variable	Perturbation variable	Correlation	P-value	Q-value
Bacterial counts	Bacterial counts	-0.09	6.9E-01	9.8E-01
Bacterial counts	Bacterial microbiome richness	0.09	6.8E-01	9.8E-01
Bacterial counts	Bacterial microbiome structure	0.04	8.8E-01	9.8E-01
Bacterial counts	Enterobacterales counts	0.04	8.7E-01	9.8E-01
Bacterial counts	Global resistome richness	-0.08	7.1E-01	9.8E-01
Bacterial counts	B-lactamasome abundance	0.40	6.8E-02	8.8E-01
Bacterial counts	B-lactamase activity	0.31	1.6E-01	9.2E-01
Bacterial counts	Phage microbiome richness	0.36	2.1E-01	9.2E-01
Bacterial counts	Phage microbiome structure	-0.28	2.2E-01	9.2E-01
Bacterial counts	Fungal load	-0.21	3.6E-01	9.4E-01
Bacterial counts	Fungal microbiome richness	-0.03	9.0E-01	9.8E-01
Bacterial counts	Fungal microbiome structure	-0.07	7.5E-01	9.8E-01
Bacterial counts	C. albicans DNA conc.	0.00	9.9E-01	>9.9E-01
Bacterial counts	Metabolome richness	0.14	5.4E-01	9.4E-01
Bacterial counts	Metabolome structure	0.17	4.6E-01	9.4E-01
Bacterial counts	Cholesterol conv. rate	-0.22	3.4E-01	9.4E-01
Bacterial counts	BA transf. capacity	0.16	4.9E-01	9.4E-01
Bacterial microbiome richness	Bacterial counts	-0.37	1.0E-01	9.2E-01
Bacterial microbiome richness	Bacterial microbiome richness	-0.12	5.8E-01	9.5E-01
Bacterial microbiome richness	Bacterial microbiome structure	-0.27	2.2E-01	9.2E-01
Bacterial microbiome richness	Enterobacterales counts	0.03	8.8E-01	9.8E-01
Bacterial microbiome richness	Global resistome richness	-0.31	1.6E-01	9.2E-01
Bacterial microbiome richness	B-lactamasome abundance	0.05	8.3E-01	9.8E-01
Bacterial microbiome richness	B-lactamase activity	0.19	4.1E-01	9.4E-01
Bacterial microbiome richness	Phage microbiome richness	-0.16	5.9E-01	9.5E-01
Bacterial microbiome richness	Phage microbiome structure	-0.29	2.1E-01	9.2E-01
Bacterial microbiome richness	Fungal load	-0.37	9.6E-02	9.2E-01
Bacterial microbiome richness	Fungal microbiome richness	-0.18	4.3E-01	9.4E-01
Bacterial microbiome richness	Fungal microbiome structure	-0.19	4.2E-01	9.4E-01
Bacterial microbiome richness	C. albicans DNA conc.	0.25	2.8E-01	9.2E-01
Bacterial microbiome richness	Metabolome richness	0.05	8.2E-01	9.8E-01
Bacterial microbiome richness	Metabolome structure	0.43	5.3E-02	8.8E-01
Bacterial microbiome richness	Cholesterol conv. rate	0.16	4.8E-01	9.4E-01
Bacterial microbiome richness	BA transf. capacity	-0.13	5.9E-01	9.5E-01
Enterobacterales counts	Bacterial counts	0.03	8.9E-01	9.8E-01

Baseline variable	Perturbation variable	Correlation	P-value	Q-value
Enterobacterales counts	Bacterial microbiome richness	0.22	3.2E-01	9.2E-01
Enterobacterales counts	Bacterial microbiome structure	-0.13	5.8E-01	9.5E-01
Enterobacterales counts	Enterobacterales counts	-0.08	7.4E-01	9.8E-01
Enterobacterales counts	Global resistome richness	-0.05	8.3E-01	9.8E-01
Enterobacterales counts	B-lactamasome abundance	0.14	5.3E-01	9.4E-01
Enterobacterales counts	B-lactamase activity	-0.10	6.5E-01	9.8E-01
Enterobacterales counts	Phage microbiome richness	-0.05	8.7E-01	9.8E-01
Enterobacterales counts	Phage microbiome structure	-0.25	2.7E-01	9.2E-01
Enterobacterales counts	Fungal load	-0.20	3.8E-01	9.4E-01
Enterobacterales counts	Fungal microbiome richness	0.47	**3.1E-02**	8.6E-01
Enterobacterales counts	Fungal microbiome structure	-0.14	5.3E-01	9.4E-01
Enterobacterales counts	C. albicans DNA conc.	0.28	2.2E-01	9.2E-01
Enterobacterales counts	Metabolome richness	-0.19	4.0E-01	9.4E-01
Enterobacterales counts	Metabolome structure	-0.23	3.2E-01	9.2E-01
Enterobacterales counts	Cholesterol conv. rate	0.08	7.3E-01	9.8E-01
Enterobacterales counts	BA transf. capacity	0.06	7.9E-01	9.8E-01
Global resistome richness	Bacterial counts	-0.30	1.9E-01	9.2E-01
Global resistome richness	Bacterial microbiome richness	-0.04	8.7E-01	9.8E-01
Global resistome richness	Bacterial microbiome structure	-0.25	2.7E-01	9.2E-01
Global resistome richness	Enterobacterales counts	-0.05	8.2E-01	9.8E-01
Global resistome richness	Global resistome richness	-0.23	3.1E-01	9.2E-01
Global resistome richness	B-lactamasome abundance	0.02	9.1E-01	9.8E-01
Global resistome richness	B-lactamase activity	0.16	4.7E-01	9.4E-01
Global resistome richness	Phage microbiome richness	-0.29	3.1E-01	9.2E-01
Global resistome richness	Phage microbiome structure	-0.29	2.0E-01	9.2E-01
Global resistome richness	Fungal load	-0.27	2.4E-01	9.2E-01
Global resistome richness	Fungal microbiome richness	-0.27	2.3E-01	9.2E-01
Global resistome richness	Fungal microbiome structure	-0.30	1.8E-01	9.2E-01
Global resistome richness	C. albicans DNA conc.	0.04	8.6E-01	9.8E-01
Global resistome richness	Metabolome richness	-0.06	7.9E-01	9.8E-01
Global resistome richness	Metabolome structure	0.50	**2.2E-02**	7.6E-01
Global resistome richness	Cholesterol conv. rate	0.25	2.7E-01	9.2E-01
Global resistome richness	BA transf. capacity	-0.02	9.2E-01	9.8E-01
B-lactamasome abundance	Bacterial counts	-0.04	8.8E-01	9.8E-01
B-lactamasome abundance	Bacterial microbiome richness	0.11	6.1E-01	9.6E-01
B-lactamasome abundance	Bacterial microbiome structure	0.10	6.5E-01	9.8E-01
B-lactamasome abundance	Enterobacterales counts	-0.07	7.7E-01	9.8E-01
B-lactamasome abundance	Global resistome richness	0.21	3.5E-01	9.4E-01
B-lactamasome abundance	B-lactamasome abundance	0.06	8.1E-01	9.8E-01
B-lactamasome abundance	B-lactamase activity	-0.08	7.1E-01	9.8E-01
B-lactamasome abundance	Phage microbiome richness	0.30	3.0E-01	9.2E-01
B-lactamasome abundance	Phage microbiome structure	-0.01	9.6E-01	9.8E-01
B-lactamasome abundance	Fungal load	0.17	4.7E-01	9.4E-01
B-lactamasome abundance	Fungal microbiome richness	0.23	3.2E-01	9.2E-01

Baseline variable	Perturbation variable	Correlation	P-value	Q-value
B-lactamasome abundance	Fungal microbiome structure	-0.15	5.1E-01	9.4E-01
B-lactamasome abundance	C. albicans DNA conc.	-0.21	3.5E-01	9.4E-01
B-lactamasome abundance	Metabolome richness	-0.30	1.9E-01	9.2E-01
B-lactamasome abundance	Metabolome structure	-0.54	**1.3E-02**	7.6E-01
B-lactamasome abundance	Cholesterol conv. rate	-0.30	1.9E-01	9.2E-01
B-lactamasome abundance	BA transf. capacity	0.13	5.7E-01	9.5E-01
B-lactamase activity	Bacterial counts	-0.13	5.8E-01	9.5E-01
B-lactamase activity	Bacterial microbiome richness	-0.01	9.6E-01	9.8E-01
B-lactamase activity	Bacterial microbiome structure	-0.48	**2.4E-02**	7.6E-01
B-lactamase activity	Enterobacterales counts	0.19	3.9E-01	9.4E-01
B-lactamase activity	Global resistome richness	-0.01	9.7E-01	9.9E-01
B-lactamase activity	B-lactamasome abundance	0.04	8.5E-01	9.8E-01
B-lactamase activity	B-lactamase activity	0.29	1.9E-01	9.2E-01
B-lactamase activity	Phage microbiome richness	-0.20	5.0E-01	9.4E-01
B-lactamase activity	Phage microbiome structure	-0.15	5.2E-01	9.4E-01
B-lactamase activity	Fungal load	-0.07	7.6E-01	9.8E-01
B-lactamase activity	Fungal microbiome richness	-0.29	2.1E-01	9.2E-01
B-lactamase activity	Fungal microbiome structure	-0.25	2.8E-01	9.2E-01
B-lactamase activity	C. albicans DNA conc.	-0.13	5.8E-01	9.5E-01
B-lactamase activity	Metabolome richness	0.04	8.6E-01	9.8E-01
B-lactamase activity	Metabolome structure	0.22	3.5E-01	9.4E-01
B-lactamase activity	Cholesterol conv. rate	0.18	4.3E-01	9.4E-01
B-lactamase activity	BA transf. capacity	0.05	8.3E-01	9.8E-01
Phage microbiome richness	Bacterial counts	-0.07	7.7E-01	9.8E-01
Phage microbiome richness	Bacterial microbiome richness	-0.20	3.8E-01	9.4E-01
Phage microbiome richness	Bacterial microbiome structure	-0.08	7.2E-01	9.8E-01
Phage microbiome richness	Enterobacterales counts	0.15	5.3E-01	9.4E-01
Phage microbiome richness	Global resistome richness	-0.04	8.5E-01	9.8E-01
Phage microbiome richness	B-lactamasome abundance	-0.14	5.4E-01	9.4E-01
Phage microbiome richness	B-lactamase activity	0.09	7.1E-01	9.8E-01
Phage microbiome richness	Phage microbiome richness	-0.23	4.4E-01	9.4E-01
Phage microbiome richness	Phage microbiome structure	0.28	2.2E-01	9.2E-01
Phage microbiome richness	Fungal load	0.02	9.3E-01	9.8E-01
Phage microbiome richness	Fungal microbiome richness	-0.29	2.2E-01	9.2E-01
Phage microbiome richness	Fungal microbiome structure	-0.27	2.4E-01	9.2E-01
Phage microbiome richness	C. albicans DNA conc.	0.17	4.7E-01	9.4E-01
Phage microbiome richness	Metabolome richness	0.24	3.1E-01	9.2E-01
Phage microbiome richness	Metabolome structure	0.37	1.0E-01	9.2E-01
Phage microbiome richness	Cholesterol conv. rate	0.25	2.8E-01	9.2E-01
Phage microbiome richness	BA transf. capacity	0.06	7.9E-01	9.8E-01
Fungal load	Bacterial counts	0.25	2.9E-01	9.2E-01
Fungal load	Bacterial microbiome richness	-0.02	9.5E-01	9.8E-01
Fungal load	Bacterial microbiome structure	0.16	4.8E-01	9.4E-01
Fungal load	Enterobacterales counts	0.32	1.6E-01	9.2E-01

**Baseline variable**	**Perturbation variable**	**Correlation**	**P-value**	**Q-value**
Fungal load	Global resistome richness	0.25	2.8E-01	9.2E-01
Fungal load	B-lactamasome abundance	0.42	6.0E-02	8.8E-01
Fungal load	B-lactamase activity	-0.35	1.2E-01	9.2E-01
Fungal load	Phage microbiome richness	-0.53	5.7E-02	8.8E-01
Fungal load	Phage microbiome structure	0.16	5.0E-01	9.4E-01
Fungal load	Fungal load	-0.55	**1.0E-02**	7.6E-01
Fungal load	Fungal microbiome richness	-0.02	9.3E-01	9.8E-01
Fungal load	Fungal microbiome structure	0.01	9.5E-01	9.8E-01
Fungal load	C. albicans DNA conc.	0.42	5.5E-02	8.8E-01
Fungal load	Metabolome richness	0.02	9.4E-01	9.8E-01
Fungal load	Metabolome structure	0.02	9.4E-01	9.8E-01
Fungal load	Cholesterol conv. rate	0.21	3.7E-01	9.4E-01
Fungal load	BA transf. capacity	0.05	8.1E-01	9.8E-01
Fungal microbiome richness	Bacterial counts	-0.03	8.9E-01	9.8E-01
Fungal microbiome richness	Bacterial microbiome richness	0.18	4.1E-01	9.4E-01
Fungal microbiome richness	Bacterial microbiome structure	0.01	9.6E-01	9.8E-01
Fungal microbiome richness	Enterobacterales counts	-0.11	6.2E-01	9.8E-01
Fungal microbiome richness	Global resistome richness	0.14	5.2E-01	9.4E-01
Fungal microbiome richness	B-lactamasome abundance	-0.06	7.9E-01	9.8E-01
Fungal microbiome richness	B-lactamase activity	0.03	8.8E-01	9.8E-01
Fungal microbiome richness	Phage microbiome richness	0.03	9.1E-01	9.8E-01
Fungal microbiome richness	Phage microbiome structure	-0.11	6.3E-01	9.8E-01
Fungal microbiome richness	Fungal load	0.08	7.3E-01	9.8E-01
Fungal microbiome richness	Fungal microbiome richness	-0.21	3.7E-01	9.4E-01
Fungal microbiome richness	Fungal microbiome structure	0.13	5.9E-01	9.5E-01
Fungal microbiome richness	C. albicans DNA conc.	-0.45	**4.1E-02**	8.8E-01
Fungal microbiome richness	Metabolome richness	-0.24	3.0E-01	9.2E-01
Fungal microbiome richness	Metabolome structure	0.11	6.4E-01	9.8E-01
Fungal microbiome richness	Cholesterol conv. rate	0.16	4.8E-01	9.4E-01
Fungal microbiome richness	BA transf. capacity	-0.21	3.7E-01	9.4E-01
C. albicans DNA conc.	Bacterial counts	-0.10	6.7E-01	9.8E-01
C. albicans DNA conc.	Bacterial microbiome richness	-0.07	7.5E-01	9.8E-01
C. albicans DNA conc.	Bacterial microbiome structure	0.03	8.8E-01	9.8E-01
C. albicans DNA conc.	Enterobacterales counts	-0.16	5.0E-01	9.4E-01
C. albicans DNA conc.	Global resistome richness	-0.31	1.7E-01	9.2E-01
C. albicans DNA conc.	B-lactamasome abundance	0.11	6.4E-01	9.8E-01
C. albicans DNA conc.	B-lactamase activity	0.16	4.7E-01	9.4E-01
C. albicans DNA conc.	Phage microbiome richness	-0.04	8.9E-01	9.8E-01
C. albicans DNA conc.	Phage microbiome structure	0.02	9.4E-01	9.8E-01
C. albicans DNA conc.	Fungal load	-0.06	8.0E-01	9.8E-01
C. albicans DNA conc.	Fungal microbiome richness	0.11	6.4E-01	9.8E-01
C. albicans DNA conc.	Fungal microbiome structure	0.17	4.5E-01	9.4E-01
C. albicans DNA conc.	C. albicans DNA conc.	0.46	**3.5E-02**	8.6E-01
C. albicans DNA conc.	Metabolome richness	0.12	5.9E-01	9.5E-01

**Baseline variable**	**Perturbation variable**	**Correlation**	**P-value**	**Q-value**
C. albicans DNA conc.	Metabolome structure	0.43	5.2E-02	8.8E-01
C. albicans DNA conc.	Cholesterol conv. rate	-0.15	5.2E-01	9.4E-01
C. albicans DNA conc.	BA transf. capacity	-0.03	9.1E-01	9.8E-01
Metabolome richness	Bacterial counts	0.00	>9.9E-01	>9.9E-01
Metabolome richness	Bacterial microbiome richness	-0.13	5.6E-01	9.5E-01
Metabolome richness	Bacterial microbiome structure	0.09	7.0E-01	9.8E-01
Metabolome richness	Enterobacterales counts	-0.22	3.2E-01	9.2E-01
Metabolome richness	Global resistome richness	0.14	5.2E-01	9.4E-01
Metabolome richness	B-lactamasome abundance	-0.08	7.2E-01	9.8E-01
Metabolome richness	B-lactamase activity	0.23	3.0E-01	9.2E-01
Metabolome richness	Phage microbiome richness	0.31	2.8E-01	9.2E-01
Metabolome richness	Phage microbiome structure	-0.14	5.4E-01	9.4E-01
Metabolome richness	Fungal load	-0.05	8.2E-01	9.8E-01
Metabolome richness	Fungal microbiome richness	-0.28	2.1E-01	9.2E-01
Metabolome richness	Fungal microbiome structure	0.00	9.9E-01	>9.9E-01
Metabolome richness	C. albicans DNA conc.	-0.40	7.6E-02	9.2E-01
Metabolome richness	Metabolome richness	-0.06	7.8E-01	9.8E-01
Metabolome richness	Metabolome structure	0.29	2.0E-01	9.2E-01
Metabolome richness	Cholesterol conv. rate	-0.16	4.9E-01	9.4E-01
Metabolome richness	BA transf. capacity	0.01	9.5E-01	9.8E-01
Cholesterol conv. rate	Bacterial counts	-0.25	2.7E-01	9.2E-01
Cholesterol conv. rate	Bacterial microbiome richness	0.30	1.7E-01	9.2E-01
Cholesterol conv. rate	Bacterial microbiome structure	-0.02	9.4E-01	9.8E-01
Cholesterol conv. rate	Enterobacterales counts	-0.08	7.3E-01	9.8E-01
Cholesterol conv. rate	Global resistome richness	-0.22	3.2E-01	9.2E-01
Cholesterol conv. rate	B-lactamasome abundance	0.21	3.4E-01	9.4E-01
Cholesterol conv. rate	B-lactamase activity	0.49	**2.0E-02**	7.6E-01
Cholesterol conv. rate	Phage microbiome richness	0.43	1.3E-01	9.2E-01
Cholesterol conv. rate	Phage microbiome structure	-0.27	2.4E-01	9.2E-01
Cholesterol conv. rate	Fungal load	-0.08	7.2E-01	9.8E-01
Cholesterol conv. rate	Fungal microbiome richness	-0.41	6.4E-02	8.8E-01
Cholesterol conv. rate	Fungal microbiome structure	0.04	8.5E-01	9.8E-01
Cholesterol conv. rate	C. albicans DNA conc.	-0.09	7.0E-01	9.8E-01
Cholesterol conv. rate	Metabolome richness	-0.04	8.5E-01	9.8E-01
Cholesterol conv. rate	Metabolome structure	0.30	1.9E-01	9.2E-01
Cholesterol conv. rate	Cholesterol conv. rate	-0.16	4.7E-01	9.4E-01
Cholesterol conv. rate	BA transf. capacity	-0.19	4.1E-01	9.4E-01
BA transf. capacity	Bacterial counts	-0.28	2.2E-01	9.2E-01
BA transf. capacity	Bacterial microbiome richness	0.18	4.1E-01	9.4E-01
BA transf. capacity	Bacterial microbiome structure	-0.16	4.9E-01	9.4E-01
BA transf. capacity	Enterobacterales counts	-0.27	2.2E-01	9.2E-01
BA transf. capacity	Global resistome richness	-0.09	6.9E-01	9.8E-01
BA transf. capacity	B-lactamasome abundance	-0.23	3.0E-01	9.2E-01
BA transf. capacity	B-lactamase activity	0.37	8.6E-02	9.2E-01

**Baseline variable**	**Perturbation variable**	**Correlation**	**P-value**	**Q-value**
BA transf. capacity	Phage microbiome richness	-0.08	8.0E-01	9.8E-01
BA transf. capacity	Phage microbiome structure	-0.52	**1.7E-02**	7.6E-01
BA transf. capacity	Fungal load	0.33	1.4E-01	9.2E-01
BA transf. capacity	Fungal microbiome richness	-0.15	5.3E-01	9.4E-01
BA transf. capacity	Fungal microbiome structure	0.13	5.8E-01	9.5E-01
BA transf. capacity	C. albicans DNA conc.	-0.60	**4.0E-03**	7.6E-01
BA transf. capacity	Metabolome richness	-0.16	4.7E-01	9.4E-01
BA transf. capacity	Metabolome structure	0.25	2.6E-01	9.2E-01
BA transf. capacity	Cholesterol conv. rate	0.23	3.1E-01	9.2E-01
BA transf. capacity	BA transf. capacity	-0.12	6.0E-01	9.6E-01

Supplementary Table S7. Baseline characteristics, maximal perturbations and maximal resiliences of the studied gut microbiota and gut components in the 22 healthy volunteers included in the CEREMI trial

Variables are colored according to their level relative to the median value, colored in white. For the baseline state of the microbiota, the green color is indicative of the high richness of the microbiota, while red indicates poor richness. For systems’ perturbation and resilience, green color indicates low perturbation and high resilience, and red color indicates high perturbation and low resilience. Subjects 3 and 16 had detectable levels of antibiotics in faeces; together with subjects 22 and 28 they had no β-lactamase activity at baseline. Conc., concentration ; Cholesterol conv. rate, Cholesterol conversion rate ; BA transf. Capacity, Bile acids transformation capacity.

	Subject ID																					
	**1**	**2**	**3**	**6**	**8**	**9**	**11**	**12**	**13**	**14**	**16**	**17**	**18**	**20**	**22**	**23**	**24**	**25**	**27**	**28**	**29**	**34**
**Baseline raw values**																						
Bacterial counts (log10 CFU/g)	11.0	11.6	11.0	11.1	11.3	11.5	11.4	11.0	11.4	11.3	11.3	11.3	11.4	11.3	10.5	11.5	11.3	11.4	11.5	11.5	11.2	11.4
Bacterial microbiome richness (MGS/g)	174	279	124	219	377	276	305	346	346	232	251	314	193	289	155	295	295	262	288	348	288	304
Enterobacterales counts (log10 CFU/g)	8.2	6.9	8.3	8.6	7.3	9.0	8.3	7.6	8.0	0.0	5.0	10.7	8.4	6.9	7.1	6.5	7.2	8.2	8.4	8.7	6.3	9.0
Global resistome richness (copies/g)	555	866	376	711	935	845	913	964	798	601	746	1008	712	902	538	989	929	888	913	1042	883	946
B-lactamasome abundance (log10)	0.18	0.14	0.15	0.14	0.12	0.15	0.12	0.13	0.13	0.14	0.16	0.15	0.15	0.14	0.15	0.14	0.14	0.14	0.14	0.15	0.14	0.14
B-lactamase activity (log10 nmol/min.g)	1.4	1.7	0.0	1.1	1.8	0.9	1.2	0.7	0.9	1.6	0.0	1.0	1.3	2.0	0.0	2.6	1.6	1.2	1.1	0.0	1.1	1.5
Phage microbiome richness (contigs/g)	988	1510	567	1296	1587	917	1472	1101	1329	1401	926	1474	744	1118	1370	1088	1219	1345	1484	1448	NA	1445
Fungal load (log10)	4E-09	NA	1E-07	2E-03	2E-04	1E-04	9E-07	8E-06	2E-05	3E-07	8E-06	1E-05	6E-10	7E-08	2E-05	6E-05	2E-06	7E-06	7E-06	6E-08	2E-06	1E-04
Fungal microbiome richness (fungal OTUs/g)	13	13	13	50	11	27	16	5	13	47	23	24	27	18	18	24	25	26	29	35	31	27
C. albicans DNA concentration (log10)	-5.3	NA	-5.8	-5.7	-3.1	-4.3	-4.5	-4.2	-3.1	-5.3	-5.8	-5.3	-5.3	-4.5	-4.7	-6.1	-4.5	-6.2	-4.4	-5.4	-5.3	-5.6
Metabolome richness (chemical species/g)	1460	1498	1362	1476	1432	1527	1403	1456	1446	1553	1572	854	1453	1537	1458	1522	1534	1491	1484	1536	1489	1483
Cholesterol conversion rate (log10)	0.0	0.9	0.0	0.1	0.9	0.9	0.6	0.8	0.8	0.9	0.8	0.7	0.9	0.9	0.0	0.9	0.8	0.5	0.8	1.0	0.9	0.7
Bile acids transformation capacity (log10)	0.9	0.8	0.6	0.8	0.6	0.8	0.9	0.8	0.8	0.9	0.6	0.8	1.0	0.7	0.3	0.9	1.0	0.9	1.0	1.0	1.0	0.9
**Perturbation : distance from baseline**																						
Bacterial counts	1.5	4.5	13.1	3.6	0.5	5.4	28.9	16.0	2.4	2.1	36.0	1.0	2.5	0.5	0.9	3.6	0.6	51.8	2.0	0.9	2.4	3.9
Bacterial microbiome richness	39.0	7.8	4.4	13.0	3.8	10.4	18.0	60.7	4.6	4.0	25.6	10.9	54.0	1.8	3.5	4.5	3.8	5.7	7.8	4.0	11.3	10.2
Bacterial microbiome structure	2.3	3.2	3.6	1.5	2.4	3.1	3.1	4.7	2.5	4.4	7.0	3.2	1.9	1.9	3.5	3.7	3.0	3.5	7.7	2.7	5.2	3.5
Enterobacterales counts	3.4	2.0	12.9	3.2	5.3	14.2	2.9	1.9	15.3	1.2	4.6	2.4	8.9	1.8	35.0	14.6	10.8	10.4	4.3	8.1	1.4	3.0
Global resistome richness	17.2	10.4	8.5	77.0	2.2	9.0	30.0	13.5	2.2	1.5	194.9	6.5	7.4	2.9	9.7	3.8	5.3	4.1	6.6	8.2	11.6	17.1
B-lactamasome abundance	1.1	3.6	1.2	5.0	7.1	23.6	6.7	4.8	16.3	5.5	14.2	9.6	30.6	5.1	3.5	14.5	1.7	5.4	3.7	2.7	9.1	10.7
B-lactamase activity	2.7	5.5	3.4	3.6	1.4	17.3	2.2	9.4	2.0	21.3	0.0	1.0	4.9	2.2	7.2	3.1	0.9	16.7	5.7	4.9	2.2	3.0
Phage microbiome richness	12.4	3.2	1.9	2.8	3.0	6.9	2.2	3.0	2.0	0.1	0.9	2.8	2.4	24.7	2.8	2.0	3.7	0.9	3.2	78.3	NA	0.4
Phage microbiome structure	2.3	1.2	1.0	1.4	1.9	1.2	1.2	2.5	1.0	1.9	1.7	2.1	1.5	1.4	1.8	1.3	1.5	1.9	2.6	1.1	NA	1.5
Fungal load	1.6	NA	1.3	0.5	2.5	0.8	0.4	0.6	5.4	1.1	1.7	1.3	2.4	3.0	4.4	1.8	2.6	1.5	5.2	1.0	1.6	1.6
Fungal microbiome richness	6.5	1.5	1.6	1.6	0.7	6.7	0.7	3.0	3.0	4.1	1.1	0.9	4.0	2.1	2.2	3.0	3.1	0.9	2.0	2.6	1.5	6.0
Fungal microbiome structure	1.0	1.0	1.1	1.1	0.9	1.1	1.0	1.3	1.2	1.2	1.1	1.1	1.1	1.1	1.1	1.1	1.0	0.9	1.2	1.1	1.2	1.0
C. albicans DNA concentration	2.4	NA	9.6	2.1	2.4	3.3	1.4	1.5	8.3	44.7	2.5	2.2	2.5	23.0	3.4	1.4	2.1	1.6	2.0	1.8	2.4	3.9
Metabolome richness	3.2	1.1	1.1	2.5	1.6	2.2	2.6	2.4	4.4	0.9	7.5	1.0	1.6	2.7	6.2	1.7	5.6	3.2	3.1	2.3	2.6	2.2
Metabolome structure	1.1	0.9	1.4	1.3	1.2	1.3	1.3	1.7	1.3	1.6	1.7	1.5	1.3	1.4	1.1	2.1	1.8	1.3	2.1	1.3	1.3	1.5
Cholesterol conversion rate	1.5	0.5	1.2	3.6	15.2	10.3	4.9	18.8	8.0	83.3	12.6	43.6	2.8	53.4	9.2	324.8	13.1	6.1	45.1	9.4	10.4	25.0
Bile acids transformation capacity	40.1	2.3	5.2	0.4	1.2	2.4	4.1	35.3	11.5	13.9	15.6	16.1	10.9	2.6	1.6	24.6	9.1	10.7	79.3	9.8	59.7	31.1
**Resilience : distance from baseline**																						
Bacterial counts	0.8	3.7	1.9	1.7	NA	2.7	0.2	9.1	0.4	1.3	0.8	0.6	0.6	1.0	1.4	0.3	0.4	21.8	1.2	0.3	1.5	1.6
Bacterial microbiome richness	0.5	5.4	1.6	3.0	0.3	3.4	0.2	7.3	0.4	1.0	0.1	1.6	8.0	0.7	0.1	0.9	0.0	0.2	0.8	0.6	3.5	0.9
Bacterial microbiome structure	0.9	2.1	1.6	1.0	1.3	2.5	1.0	1.4	1.3	1.2	4.6	1.4	1.3	0.9	2.4	0.9	1.4	1.7	1.6	1.0	2.5	1.0
Enterobacterales counts	0.5	0.5	0.1	0.7	1.0	0.0	0.6	0.2	6.0	0.7	0.7	0.9	1.6	0.4	2.8	2.1	0.6	2.6	0.0	0.3	0.0	0.7
Global resistome richness	1.7	7.3	1.4	33.5	0.4	1.4	1.2	0.5	0.1	1.3	9.1	1.0	0.1	0.3	0.2	0.8	0.3	0.3	1.0	0.1	3.9	1.7
B-lactamasome abundance	0.3	0.2	0.1	0.2	1.6	6.9	0.6	0.2	1.7	0.3	1.7	0.8	6.4	0.5	1.2	1.8	0.1	0.6	0.7	0.1	0.0	1.5
B-lactamase activity	2.1	2.7	0.0	0.0	0.9	1.7	0.2	3.9	0.6	9.0	0.0	0.4	2.1	0.8	0.0	0.6	0.2	1.9	0.2	2.3	1.2	0.3
Phage microbiome richness	NA	NA	2.7	0.3	NA	2.3	0.8	0.1	2.1	NA	NA	0.1	1.3	13.7	NA	0.7	0.5	NA	0.7	14.8	NA	0.6
Phage microbiome structure	0.9	1.2	1.1	1.2	1.7	1.0	1.0	1.1	0.9	1.2	1.4	1.3	1.1	1.0	2.0	0.7	1.0	1.3	1.2	0.7	NA	0.8
Fungal load	1.0	NA	0.8	0.0	0.3	0.2	0.9	0.9	0.1	1.2	0.4	0.3	1.3	0.6	3.5	0.5	0.2	0.6	1.2	0.7	1.4	0.7
Fungal microbiome richness	2.0	NA	0.5	0.5	0.2	1.0	1.1	1.1	0.5	0.3	0.5	0.3	0.6	0.3	0.4	0.2	0.2	0.3	0.4	0.3	0.1	4.7
Fungal microbiome structure	0.9	NA	0.9	1.0	0.7	0.9	0.9	1.0	1.1	1.0	0.9	0.8	0.9	0.8	0.9	1.0	0.8	0.7	1.1	0.8	1.0	0.7
C. albicans DNA concentration	0.3	NA	1.3	0.6	1.9	1.9	0.6	0.4	4.9	0.0	0.2	0.9	0.0	3.0	0.8	0.4	0.1	0.0	1.0	0.3	0.0	0.4
Metabolome richness	0.8	NA	0.3	0.2	0.4	0.5	1.7	0.1	1.7	0.2	0.4	0.7	0.1	1.9	1.7	1.4	0.0	1.5	1.4	0.4	1.1	0.2
Metabolome structure	0.8	NA	0.8	0.8	1.0	1.1	1.2	1.1	0.8	1.0	0.9	0.9	0.8	1.1	0.8	1.0	1.2	0.9	1.3	1.0	1.2	0.8
Cholesterol conversion rate	0.2	NA	0.8	1.1	0.2	0.0	0.1	0.2	0.3	0.2	0.1	1.7	0.0	0.3	0.1	0.4	0.8	0.2	0.9	0.1	2.5	0.7
Bile acids transformation capacity	0.8	NA	0.8	0.1	0.6	0.8	2.5	0.2	0.1	0.2	0.8	0.1	0.1	0.3	1.2	0.9	0.2	0.1	1.1	0.4	0.0	2.7
